# Supplementary material for: Developing the evidence base for evaluating dementia training in NHS hospitals (DEMTRAIN): a mixed-methods study protocol
Source: BMJ Open. 2020 Jan 14;10(1):e030739. doi: 10.1136/bmjopen-2019-030739 (PMC7045160; doi:10.1136/bmjopen-2019-030739)
Supplement: Author's manuscript [file bmjopen-2019-030739.draft_revisions.pdf]

BMJ Open is committed to open peer review. As part of this commitment we make the peer review history of every article we publish publicly available.

When an article is published we post the peer reviewers' comments and the authors' responses online. We also post the versions of the paper that were used during peer review. These are the versions that the peer review comments apply to.

The versions of the paper that follow are the versions that were submitted during the peer review process. They are not the versions of record or the final published versions. They should not be cited or distributed as the published version of this manuscript.

BMJ Open is an open access journal and the full, final, typeset and author-corrected version of record of the manuscript is available on our site with no access controls, subscription charges or pay-per-view fees (http://bmjopen.bmj.com).

If you have any questions on BMJ Open's open peer review process please email info.bmjopen@bmj.com

BMJ Open

Developing the evidence base for evaluating dementia training in NHS hospitals (DEMTRAIN): a mixed methods study protocol

Journal:	*BMJ Open*
Manuscript ID	bmjopen-2019-030739
Article Type:	Protocol
Date Submitted by the Author:	29-Mar-2019
Complete List of Authors:	Ahmed, Faraz; Lancaster University Faculty of Health and Medicine, Health Research Division<br>Morbey, Hazel; Lancaster University Faculty of Health and Medicine, Health Research Division<br>Harding, Andrew; Lancaster University, Division of Health Research, Faculty of Health and Medicine<br>Reeves, David; University of Manchester<br>Swarbrick, Caroline; Lancaster University Faculty of Health and Medicine, Health Research Division<br>Davies, Linda; University of Manchester<br>Hann , Mark ; University of Manchester<br>Holland, Fiona; University of Manchester<br>Elvish, Ruth; University of Manchester<br>Leroi, Iracema; University of Manchester<br>Burrow, Simon; University of Manchester<br>Burns, Alistair; University of Manchester<br>Keady, John; University of Manchester<br>Reilly, Siobhan; Lancaster University, Division of Health Research
Keywords:	Dementia < NEUROLOGY, Hospitals, Dementia training, Mixed methods, EDUCATION & TRAINING (see Medical Education & Training)

SCHOLARONE™
Manuscripts

Title: Developing the evidence base for evaluating dementia training in NHS hospitals (DEMTRAIN): a mixed methods study protocol

Authors:

Faraz Ahmed*
Hazel Morbey*
Andrew Harding*
David Reeves**
Caroline Swarbrick*
Linda Davies**
Mark Hann**
Fiona Holland**
Ruth Elvish**
Iracema Leroi**
Simon Burrow**
Alistair Burns**
John Keady**
Siobhan T. Reilly*

* Lancaster University, Division of Health Research, Lancaster University, Lancaster LA1 4YG

** University of Manchester, Jean McFarlane Building, Oxford Road, Manchester M13 9PL

Corresponding Author:

Siobhan T. Reilly, Lancaster University, Division of Health Research, Lancaster University, Lancaster LA1 4YG **Email:** s.reilly@lancaster.ac.uk **Tel:** + 44(0)1524594367

For peer review only

Abstract:

Introduction: Around 70 percent of acute hospital beds in the UK are occupied by older people, approximately 40 percent of whom have dementia. Improving the quality of care in hospitals is a key priority within national dementia strategies. Limited research has been conducted to evaluate dementia training packages for staff, and evaluation of training often focuses on immediate, on-the-day training feedback and effects.

Objectives: Our study aims to answer two research questions:

(1) How do variations in content, implementation and intensity of staff dementia training in acute hospitals in England relate to health service outcome/process measures and staff outcomes?

(2) What components of staff dementia training are most strongly related to improved patient and staff outcomes?

Methods and analysis: Using the principles of programme theory, a mixed method study will be used to identify mechanisms and the interactions between them, as well as facilitators and barriers to dementia training in hospitals. We will use existing data, such as Hospital Episode Statistics, alongside two surveys (at hospital and staff level).We will recruit up to 193 acute hospitals in England to participate in the hospital level survey. We aim to recruit up to 30 staff members per hospital, from a random sample of 24 hospitals. In addition, we will explore the cost effectiveness of dementia training packages and carry out an in-depth case study of up to six hospitals.

Ethics and dissemination: The study has been reviewed and approved by the Faculty of Health and Medicine Research Ethics Committee (FHMREC 17056) and HRA (IRAS ID 242166: REC reference 18/HRA/1198). We plan to develop both standard (e.g. academic publications, presentations at conferences) and innovative (e.g. Citizen Scientist web portals, online fora, links with hospitals and third sector organisations) means of ensuring the study findings are accessible and disseminated regionally, nationally and internationally.

For peer review only

Article Summary

Strengths and limitations of this study

- This study will use a range of existing datasets (including Hospital Episode Statistics, and National Audit of Dementia's Organisational Checklist) and mixed methods approaches to make recommendations about dementia training in acute hospital settings.
- Using the principles of programme theory, we will expand on mechanisms and the interactions between them, as well as facilitators and barriers to dementia training in the hospitals.
- This study aims to explore the long term impact of dementia training (if any) on health service outcomes/ process measures (e.g. length of stay, emergency re-admissions and mortality) and staff outcomes (e.g. confidence, staff strain, knowledge of dementia).
- Given the multiple initiatives and dementia policies employed in hospitals across England, it will be difficult to establish the effects of dementia training directly on patient and staff outcomes.

Keywords:

Dementia, Hospitals, Dementia training, Mixed methods

For peer review only

Introduction

Improving the quality of care in general hospitals continues to be one of the key priorities within national dementia strategies [1-3]. Acute NHS Trusts provide a range of services, including accident and emergency departments, inpatient and outpatient medicine and surgery and in some cases very specialist medical care [4]. They provide secondary care, ranging from relatively small district hospitals to large city teaching hospitals in England. The term acute, generally refers to physical illnesses and conditions, which are usually short-term and require diagnostic tests, treatment and follow-up care [4 5]. At any one time, around 70 percent of acute hospital beds are occupied by older people, 40% of whom have dementia [6-8]. Dementia awareness and care training is widely thought to increase staff competencies [9 10], although types of training and how effectiveness is assessed is highly variable.

Since 2013, Health Education England (HEE) has overseen increases in the provision of foundational-level dementia training [11 12]. However, limited research has so far been conducted to evaluate dementia training packages. Evaluation tends to focus on immediate, on-the-day training feedback and effects. Moreover, training for staff in any care setting is seldom evaluated for its impact on the care of people living with dementia and for long term effects and impact on staff [13 14]. Given the high variability of dementia training packages [10], there is a need to better understand the current evidence and context of dementia training that is provided in acute NHS hospitals. This study will explore the complex workings of dementia training, including exploring relationships with patient and staff outcomes. This mixed methods study aims to address two overarching research questions:

1) How do variations in content, implementation and intensity of dementia training in hospitals in England relate to health service outcome/process measures (e.g. length of stay, emergency re-admissions and mortality) and staff outcomes (e.g. confidence, staff strain, knowledge of dementia)?
2) What components of dementia training are most strongly related to improved patient and staff outcomes?

The findings will lead to a set of specific recommendations and guidance about which elements of training packages are more effective and appropriate for use in acute hospitals.

Methods and analysis

The three phases of the study are:

- **Phase 1** Mapping the evidence base for dementia training in hospitals
- **Phase 2** Cohort study to assess differences in acute NHS hospital outcomes for people living with dementia compared to those without, and the impact of training methods on patient outcome.
- **Phase 3** Multiple hospital case studies of staff dementia training and care in acute NHS hospitals.

For peer review only - http://bmjopen.bmj.com/site/about/guidelines.xhtml

Our mixed methods study will utilise the principles of programme theory [15] to identify mechanisms and the interactions between them, as well as the facilitators and barriers to dementia training in the hospital. DEMTRAIN, one of eight work programmes within the Neighbourhoods and Dementia Study (funded by the ESRC/NIHR), is developing the evidence base for evaluating dementia training in acute NHS hospitals.

Phase 1: Mapping the evidence base for dementia training in Hospitals

Aims:

1. To review the current evidence base for staff dementia training in hospital settings
2. To map the variation in staff dementia training currently provided to acute hospital staff in England
3. To assess the impact of differences in staff dementia training upon staff knowledge, practice, organisational culture and staff strain in acute NHS hospitals.

Methods:

Phase 1 will involve three main elements:

I. We will conduct **a systematic review** of the evidence base for training hospital staff in dementia awareness and care exploring both the quality and effectiveness of training programmes. This will be achieved by conducting a critical interpretive synthesis [16], which will form the basis of a logic model using the principles of programme theory to identify mechanisms, interaction, facilitators and barriers to dementia training in hospital settings. The protocol for this element of phase 1 is registered on PROSPERO and is not outlined here [17].

II. We will conduct an **organisational surveys** in acute NHS hospitals in England (approx. 193 hospitals will be approached using CCQI National Audit of Dementia (NAD) sample frame contact list) to identify training strategies; establish the presence, frequency, duration, characteristics and format of training programmes; quality of training and resources utilised in developing training. We will also explore what other initiatives are implemented (e.g. having a dementia champion, active participation in Butterfly scheme, Dementia Friends, carers allowed on wards at meal times).

III. We will conduct a survey of staff (**hospital staff survey**) to collect data on the extent of training, knowledge, attitudes, staff strain, organisational culture and satisfaction in caring for patients living with dementia. We aim to recruit 24 hospitals randomly sampled from stratified lists of hospitals scoring low, mixed and high across three main domains of dementia care (governance, staff training and patient care) derived from the NAD in 2016).

Recruitment for organisational survey in acute NHS hospitals in England

We will use NAD data to identify potential hospitals for the hospital organisational survey. Our preliminary discussions with our advisory group (including representatives from NAD who are involved in similar surveys, and local Dementia Leads who are hospital staff responsible for

For peer review only - http://bmjopen.bmj.com/site/about/guidelines.xhtml

ensuring that their hospital is aligned with NHS Trust and National dementia strategies, and involved in developing and implemented dementia training in their hospitals), suggests that in most cases Dementia Leads at each hospital will have access to the data needed to complete the organisational level survey. However, given the segmented and large variations in recording systems for dementia training across Hospitals in England, the information needed to complete the survey may be held in other departments in the hospital, such as other IT or human resources (HR) systems. On this basis, we will have multiple survey collection approaches (i.e. online and telephone completion), with researcher-led support to ensure high quality data and completion. Figure 1 presents the hospital recruitment process for the Organisational Survey.

INSERT FIGURE 1

Dementia Leads will be responsible for completing the data. However, in cases where the information needed to complete the Hospital Organisational Survey is not directly available to the Dementia Leads, the Dementia Leads will act as Location Navigators and will direct requests to complete the survey to the appropriate person in the acute NHS hospital.

Before starting the organisational survey, participants will be asked if they have discussed participation in this study with appropriate members in their organisation, and that their hospital and or Trust has given assent to participate.

Recruitment for staff survey in two specific wards in acute NHS hospitals in England

Using the NAD returns for 2016, we combined across sets of related question items to construct performance scores for each hospital on three main domains of care for inpatients with dementia: governance; staff training; and patient care (policies/processes concerning nutrition, discharge, assessment, communication and carers' rating of patient care). We then identified subgroups of hospitals scoring high, low and mixed across the three domains. Within each of these subgroups hospitals were randomly sampled for invitation into the survey, with the objective of recruiting 8 hospitals from within each subgroup (24 in total).

We will survey different types of staff members working on two contrasting acute wards where dementia is expected to be prevalent:

- **Ward 1: Elderly Care /Older people's ward** (if the acute NHS hospital does not have these wards, an **orthopaedic ward** will be selected instead)

- **Ward 2: General Surgery or surgical ward** (if the acute NHS hospital does not have these wards, an **oncology ward** will be selected instead)

For peer review only - http://bmjopen.bmj.com/site/about/guidelines.xhtml

We will recruit staff from five categories in each acute NHS hospital:

1) Doctors
2) Nurses
3) Health Care Assistants
4) Allied healthcare professionals (e.g. physiotherapists, dieticians)
5) Support staff in the hospital (e.g. housekeepers, porters, receptionists)

In view of our member involvement (alternatively termed as Patient and Public Involvement) and Advisory Group feedback, our sample will focus on staff categories with more frequent interactions with patients living with dementia. It is thought that the above staff groups may be more likely to affect patient-related processes of care and outcomes. Staff will be sent electronic or postal copies of the staff survey depending on individual preference.

Location Navigators and local Clinical Research Networks (CRNs) will help identify 15-30 participants from each hospital. The research team will inform location navigators and CRN to recruit staff in a particular ward on a particular day and to select a random sample from ward/hospital staff lists/registers (e.g. every fourth staff member on the list will be approached). However, this may not be possible in the absence of staff lists/registers. Data on representativeness of the sample in relation to the ward will also be collected (i.e. estimated number of staff on ward on a given day, in comparison to the number of surveys completed).

Phase 2: Cohort study to assess differences in acute NHS hospital outcomes for people living with dementia compared to those without, and the impact of training methods on patient outcome

Aim:

To determine the types and elements of training that are most strongly associated with hospital outcomes for people living with dementia, including length of stay, emergency readmissions (ERs within 30 days of prior discharge), and mortality (death within 30 days of discharge) for older people.

Methods:

The primary source of information on hospital outcomes will be the Hospital Episode Statistics (HES) database, which holds routinely collected information on all inpatient spells in England and Wales. Outcomes for patients with a diagnosis of dementia will be compared to outcomes for patients without a diagnosis, with a focus on how hospital training scores relate to the difference in outcomes between these two groups. Comparisons will be made separately for the financial years 2010/11, 2012/13 and 2016/17 to coincide with data on staff training and other dementia initiatives in hospitals collected under the NAD (see below). Patients living with dementia will be defined as those with a diagnosis of dementia reported in the hospital record

For peer review only - http://bmjopen.bmj.com/site/about/guidelines.xhtml

at any time over the previous 5 years; patients without a diagnosis will be used as controls. This approach has been used in several previous studies (e.g. Care Quality Commission, State of health care and social care in England in 2012/13 Technical Annex 3 [18]) and the rate of misclassification is believed to be acceptably small [19]. Corresponding NAD data for 2010/11, 2012/13 and 2016/17 will be used to construct hospital-level scores on a number of key domains of dementia care, in particular governance, staff training and patient care.

Details of additional secondary research data

We will use other relevant secondary data where appropriate. In addition, we will develop a number of collaborations and explore other sources of data, such as data already available in the public domain or routinely collected as part of service evaluation. These may include further information on care, details and rates of staff training and patient experience among people living with dementia.

Phase 3: Multiple hospital case studies of staff dementia training and care in acute NHS hospitals

Aims:

1) To determine the content, implementation and intensity of training in contrasting hospitals and to explore the acceptability and feasibility of training to a range of hospital staff.

2) To identify barriers and facilitators for the implementation, and sustainability of staff dementia training in acute NHS hospitals.

3) To identify lessons about the implementation of staff dementia training that might be applicable in other care settings.

Methods:

Case study approaches in health research tend to involve multiple methods and offer pragmatic and appropriate approaches to provide an in-depth and strong focus on being able to report on how findings are contingent on context [20 21]. Observational methods further enhance the possibility for insights in case study research [22]. Our design includes multiple data collection approaches, including observations, focus group and interviews within each case study hospital. This phase of the study is predominately qualitative with data collection from multiple sources with a range of stakeholders within each hospital. We will utilise the principles of programme theory and realist approach to identify theories of change with respect to training initiatives in different hospital contexts [23 24]. For instance to identify mechanisms and the interactions between them, as well as facilitators and barriers to dementia training in the hospital and its related outcomes. Programme theory, within the theory-driven evaluation field, refers to a variety of ways of describing and evaluating a programme or intervention (such as staff dementia training) by developing an understanding of the causal modal that connect the inputs and activities of an intervention to its outcomes [15].

For peer review only - http://bmjopen.bmj.com/site/about/guidelines.xhtml

Using an organizational case study design we will explore the implementation of training practices in hospitals [25]. Using data from Phase 1, we will select up to six contrasting hospitals based on a number of factors related to the implementation of training such as: intensity, duration coverage of training; geography (Sustainability and Transformation Plans region and hospital type) (see table 1). A series of semi-structured interviews and focus groups will be conducted with a purposive sampling strategy to recruit staff with different levels of training and experience (including, doctors, nurses, support and domestic staff). These interviews and focus groups will explore expectations and experiences of staff dementia training among hospital staff. In addition, researchers will observe staff activity on wards at up to two shift changeovers. Observations will focus on administrative/general duties and the work environment related to dementia care. Researchers will visit case study hospitals over three days, guided by the Edbrooke-Childs *et al*. (2018) Huddle Observation Tool for inpatient clinical wards (please see below for more details on observations of staff on ward).

Insert Table 1 here

Recruitment and data collection in case study sites

Case study site visits will take place over a three-day period to undertake interviews, focus groups and observational data collection (for more detail see figure 2, and Table 1).

Insert Figure 2 here

Observations of staff on ward

We will observe staff activity on wards at up to two staff shift changeovers, focusing on their administrative/general duties and the work environment. For example, before the start of the shift, or observation of domestic staff and porters at meals times. The observations will be guided by the Edbrooke-Childs *et al*. [26] Huddle Observation Tool for inpatient clinical wards. This approach explores four main areas on the ward in relation to staff general/administrative duties:

1. 'Risk management' (e.g. 'Were there opportunities to identify risks and come up with concrete plans for these risks?')
2. 'Structure' (e.g. 'Did the activities have a clear structure?')
3. 'Collaborative culture' (e.g. 'Did everyone have the opportunity to contribute and were all points of view respected?')
4. 'Environment' (e.g. Physical and emotional environment)

Researchers will only make observation notes during a visit. Observations will not be connected to a specific individual or staff member. It will be made clear and staff will be reassured, that the aim of observation of administrative and general duties is to identify best practice rather than highlight faults. Permissions to conduct the observation on a ward will be

For peer review only - http://bmjopen.bmj.com/site/about/guidelines.xhtml

sought from the Location Navigator (e.g. ward managers/Dementia Leads), except for in a public space (e.g. main entrance or reception area). All researchers will have appropriate approvals from local R&D and HRA, including relevant training (e.g. research passport and good clinical practice).

Interviews and focus groups of hospital staff

We will aim to conduct two focus groups per hospital (total: 12 groups) with a range of staff. Location navigators and CRN will help identify staff and distribute information sheets and consent forms. Five participants from groups designated 'patient facing' and 'patient supporting' will be selected purposively, as to allow a maximum of 10 participants per focus group:

1. **Patient facing:** Doctors, nurses, health care assistants, allied health professionals, including outpatient, A&E, X-rays, dietitians, frailty ward staff, student nurses.
2. **Patient supporting:** Social Care, Voluntary (Community and Hospital), Discharge Staff, Patient Advice and Liaison Service staff, Car Park, & Security Staff, Porters, and other Domestic and support staff

We will conduct up to five interviews per hospital with a range of staff, including:

- Director of Nursing
- Dementia Lead or Champion
- Bank/Externally Contracted staff
- Manager/Health Administrator
- Student Nurse

We estimate each interview and focus group to last on average an hour and will be audio recorded on encrypted devices. The group will be shown a summary of their hospital's performance in seven areas, which is currently available from NAD. This will encourage greater engagement and interaction between the participants and serve to prompt discussion. We expect the focus groups to take place at the hospitals.

Analysis of data

Quantitative analysis

We will use Stata to analyse the quantitative data collected. The main focus will be to examine how hospital training scores relate to the difference in outcomes between patients with a known diagnosis of dementia, compared to outcomes for patients without a known diagnosis. We will develop a number of statistical models to determine the types and elements of training that are most strongly associated with changes in the LoS, emergency readmissions within 30 days of prior discharge, outcomes related to the care received in the hospital for people living with dementia and mortality (death within 30 days of discharge) for older people. We will use NAD data (2010/11, 2012/13 and 2016/17) to construct hospital-level scores on a number of

For peer review only - http://bmjopen.bmj.com/site/about/guidelines.xhtml

key domains of dementia care, in particular governance, staff training and patient care. Statistical models will be fitted to assess relationships between scores on the different domains of dementia care and differences in outcomes between people living with dementia compared to those without, after controlling for a range of confounding variables including patient demographics, pre-existing health and reasons for hospital admission. A further analysis will examine relationships between outcomes and dementia-care scores derived from our own hospital organisational survey in 2016/17 only.

Qualitative analysis

A thematic analysis [27] will be undertaken to identify emerging themes (including a-priori themes, in view of the literature and current dementia policies) in relation to case study interviews, focus groups and observations. Data will be imported, coded and managed using the qualitative data analysis software tool (NVivo v11). The analysis process will be guided by our programme theory that is being developed as part of a systematic review (i.e. critical interpretive synthesis) of the current evidence-base on dementia training in hospital settings [17].

Economic analysis

A combined decision analytic/simulation model will be developed from the Phase 1 and 3 data and review of the literature. The economic evaluation will use data from Phases 1-3 to develop the model structure and explore the costs, outcomes and relative cost effectiveness of alternative training interventions from the perspective of health and social care services and patients. The primary measure of benefit will be the Quality Adjusted Life Year (QALY) measure. Incremental cost-effectiveness ratios (ICERs) will be calculated. Cost-effectiveness acceptability curves, the probability of whether training is cost-effective, and net benefit statistics will be estimated to explore uncertainty in the data. One and multi-way sensitivity analyses will be used to explore the impact of alternative training methods over alternative time horizons (e.g. 5 and 10 years), patient populations and settings, alternative data sources and range of costs are included. This will include assessment of the impact of barriers and facilitators to implementing training and staff participation and compliance with training.

DEMTRAIN Patient and Public Involvement and Co-research model.

There is increasing recognition that inclusion of people living with dementia beyond the realm of 'participant' to involvement in all areas of the research process in ways which are both personally meaningful and relationally supportive is imperative [28]. The CO-research INvolvement and Engagement in Dementia or COINED model [29] forms part of the work of the Neighbourhoods and Dementia Study and is a unique and positive feature of the DEMTRAIN study. The term 'co-researcher' reflects collaborative, co-operative and community-based partnership between groups of people living with dementia, academic researchers and service providers. We have involved people living with dementia and their care partners in a range of areas including:

- development of the organisation and staff level survey,
- development of qualitative interview and focus group guide,
- regular feedback on study design and implementation.

For peer review only - http://bmjopen.bmj.com/site/about/guidelines.xhtml

Care partners' is the term selected by Co-research involvement groups of our Neighbourhoods Study, to refer to those who look after, support and care for someone living with dementia, in a non-professional, non-paid capacity [30]. This may be a family member, friend or neighbour.

The qualitative analysis of case study data provides an additional opportunity to bring in the perspectives of people living with dementia and their care partners at the analysis stage as co-researchers. We, therefore, propose various activities that will help in the interpretation of the data/findings and help us bring their valuable perspectives:

- Co-researchers will inform the development of the DEMTRAIN programme theory, that is part of a systematic review (i.e. critical interpretive synthesis) of the current evidence-base on dementia training in hospital settings, and that serves as theoretical framework to our study.
- Co-research observational visits to hospital public spaces (e.g. main reception and public eating areas), in up to six sites, with researchers who are involved in qualitative data collection for case studies.
- Co-researchers will review various anonymised iterations of the analysed summary/data.

All co-researchers will receive pre- and post-visit briefing before each hospital visit to discuss any issues or challenges concerning their observations. They will be accompanied by a researcher at all times and, if they wish, an informal care partner may escort them during the visit. Given the nature of the research and data collection, we envisage no risk to researchers. However, Work Programme 8 (WP8) provides a well-being service for all Programme staff, co-researchers and members working within the Neighbourhoods Study. WP8 is led by Dr Ruth Elvish, clinical psychologist, who provides this confidential support service.

Ethics and dissemination

The study has been reviewed and approved by the Faculty of Health and Medicine Research Ethics Committee (FHMREC 17056) and HRA (IRAS ID 242166: REC reference 18/HRA/1198). We plan to develop both standard (e.g. academic publications, presentations at conferences) and innovative (e.g. Citizen Scientist web portals, online fora, links with hospitals and third sector organisations) means of ensuring the study findings are accessible and disseminated regionally, nationally and internationally.

Ethical considerations

Given the nature of the topics and methods adopted, the study poses a low risk. However, we understand that hospital staff may be anxious or fearful of reprisal from other colleagues or hospital management when completing a survey, such as our hospital staff survey, that explores their skills, competencies and satisfaction in regards to dementia training (particularly if the training is developed and provided by their own hospital). To manage these risks all participant answers will be separate from any identifiable information, and we will adhere to Lancaster University guidelines on confidentiality and anonymity of participants. A key issue

For peer review only - http://bmjopen.bmj.com/site/about/guidelines.xhtml

concerning staff observation is whether the individual concerned is 'identifiable' from the information collected (i.e. in the observation notes). However, we will exclude any identifiable information in the notes and focusing only on administrative/general duties and the work environment.

Information provided to staff invited to complete a survey will ensure they are aware of this anonymity and that they are under no obligation to take part in the study. Participants will also be made aware that they are free to withdraw from a survey/interview/focus group if they wish. Staff will be informed that once a survey/interview/focus group is complete they have two weeks to withdraw the survey by submitting a request to the research team. Consent to participate in the staff survey will be taken as granted on completion of the survey. Before each interview and focus group, a researcher will go through the study information sheet and seek informed consent for each participant. Staff will be made aware that they are free to leave an interview or focus group if they wish. The consent forms will ensure that staff are aware that their participation is confidential, and that we will be audio recording the discussions.

Any quotes or reporting will not identify individual staff or their hospital. Audio files will be stored safely and securely in line with university and our study data management plan (DMP). For instance, all digital data (i.e. audio recordings) will be stored on encrypted audio recorders (and backed up at the earliest opportunity onto encrypted hard drives and subsequently onto secure University servers. Only the research team and those involved in transcribing the data will have access to the raw data. All participant information will be stored in locked cabinets.

All staff participating in the study (i.e. staff survey, Interview and focus group participants) will be offered a £10 voucher as acknowledgement of their contribution and time. Similarly, CPD certificates for participation in research will be provided to all staff members who indicate that they wish to receive one. As thanks for participation in the study, we will offer a Dementia Clock to the hospital wards taking part in the staff survey (up to two Dementia Clocks per hospital). Summary reports based on the primary data collection and secondary data (i.e. HES and NAD data) will be made available to all hospitals who complete the hospital organisational survey and indicate that they wish to receive one. Given individual staff responses will be confidential this summary will not include information relating to the staff survey. Completed organisational surveys will be entered into a prize draw for up to 10 hospitals to receive £500 for either equipment that enhances the hospital experience for patients living with dementia or a dementia-related charitable donation.

Strengths and limitations

This study will use the principles of programme theory to expand on mechanisms and the interactions between them, facilitators and barriers to dementia training in acute hospitals in England. One of the key strengths of this study is that it uses a range of existing datasets (including Hospital Episode Statistics and National Audit of Dementia and mixed methods approaches to make recommendations in relation to dementia training in acute hospital settings. Unlike previous studies that have focused on immediate, on-the-day training feedback and effects of dementia training in hospital settings [9 10], our proposed study will

For peer review only - http://bmjopen.bmj.com/site/about/guidelines.xhtml

attempt to explore the longer-term impact of dementia training. Given the multiple initiatives and dementia policies employed in hospitals across England, it will be challenging to establish the effects of dementia training directly on patient and staff outcomes. Nonetheless, we will use statistical models and mixed methods approaches to adjust and triangulate our findings.

Another key strength of our study is that across each of the study phases, we will continue to facilitate the involvement of people living with dementia as co-researchers, guided by the COINED model of co-research. As well as, consultations with our collaborators and key stakeholders involved in similar form of studies, such as What Works' in Dementia Education and Training evaluation (http://www.leedsbeckett.ac.uk/pages/what-works/), National Audit of Dementia (NAD), and local Clinical Research Network (CRN). Our project partner, Advancing Quality Alliance (AQuA) an improvement body working across the NHS in the North West will also be consulted to aide recruitment and dissemination of the findings.

For peer review only

References

1. Department of Health. Prime Minister's challenge on dementia 2020. London, 2015.

2. Alzheimer's Society. Building dementia-friendly communities: A priority for everyone, 2013.

3. Department of Health. Living well with dementia. A national dementia strategy, 2009. London: Department of Health 2009.

4. NHS. NHS authorities and trusts 2016 [Available from: https://www.nhs.uk/using-the-nhs/about-the-nhs/nhs-authorities-and-trusts/ accessed 6 March 2019.

5. Northern Devon Healthcare NHS Trust. Definitions 2017 [Available from: https://www.northdevonhealth.nhs.uk/have-your-say/current-consultations-and-engagement-opportunities/successregime/acute-services-review/definitions/ accessed 6 March 2019.

6. Department of Health. Older People: National Service Framework for Older People. 2001

7. King's College London, The London School of Economics, Alzheimer's Society. Dementia UK, 2007.

8. Prince MJ, Knapp M, Guerchet M, et al. Dementia UK: Update Second edition. second ed. London, 2014.

9. Surr CA, Gates C, Irving D, et al. Effective Dementia Education and Training for the Health and Social Care Workforce: A Systematic Review of the Literature. *Review of educational research* 2017;87(5):966-1002. doi: 10.3102/0034654317723305 [published Online First: 2017/07/31]

10. Scerri A, Innes A, Scerri C. Dementia training programmes for staff working in general hospital settings – a systematic review of the literature. *Aging & Mental Health* 2017;21(8):783-96. doi: 10.1080/13607863.2016.1231170

11. Department of Health. Delivering high quality, effective, compassionate care: Developing the right people with the right skills and the right values. A mandate from the Government to Health Education England: April 2013 to March 2015. London: Crown, 2013.

12. Department of Health. Delivering high quality, effective, compassionate care: Developing the right people with the right skills and the right values. London: Crown, 2014.

13. CQC. Cracks in the Pathway, 2014-People's experiences of dementia care as they move between care homes and hospitals. London, 2014.

14. Spector A, Orrell M, Goyder J. A systematic review of staff training interventions to reduce the behavioural and psychological symptoms of dementia. *Ageing Research Reviews* 2013;12(1):354-64. doi: http://dx.doi.org/10.1016/j.arr.2012.06.005

15. Rogers PJ. Using Programme Theory to Evaluate Complicated and Complex Aspects of Interventions. *Evaluation* 2008;14(1):29-48. doi: 10.1177/1356389007084674

16. Woods MD, Kirk MD, Agarwal MS, et al. Vulnerable groups and access to health care: a critical interpretive review. *National Coordinating Centre NHS Service Delivery Organ RD (NCCSDO) Retrieved May* 2005;27:2012.

For peer review only - http://bmjopen.bmj.com/site/about/guidelines.xhtml

17. Ahmed F, Morbey H, Harding A, et al. A critical interpretive synthesis (CIS) review of the current evidence base for dementia training in hospitals. *PROSPERO* 2018. http://www.crd.york.ac.uk/PROSPERO/display_record.php?ID=CRD42018088625 (accessed 14 February 2018).

18. CQC. State of health care and social care in England in 2012/13. Technical Annex 3. London: The Stationery Office 2013.

19. Sommerlad A, Perera G, Singh-Manoux A, et al. Accuracy of general hospital dementia diagnoses in England: Sensitivity, specificity, and predictors of diagnostic accuracy 2008-2016. *Alzheimer's & dementia : the journal of the Alzheimer's Association* 2018;14(7):933-43. doi: 10.1016/j.jalz.2018.02.012 [published Online First: 2018/04/29]

20. Carolan CM, Forbat L, Smith A. Developing the DESCARTE Model: The Design of Case Study Research in Health Care. *Qualitative Health Research* 2016;26(5):626-39. doi: 10.1177/1049732315602488

21. Walshe C. The evaluation of complex interventions in palliative care: An exploration of the potential of case study research strategies. *Palliative Med* 2011;25(8):774-81. doi: 10.1177/0269216311419883

22. Morgan SJ, Pullon SR, Macdonald LM, et al. Case Study Observational Research: A Framework for Conducting Case Study Research Where Observation Data Are the Focus. *Qual Health Res* 2016;27(7):1060-68. doi: 10.1177/1049732316649160

23. Pawson R, Tilley N. Realistic Evaluation. . London: Sage 1997.

24. Clarke DJ, Godfrey M, Hawkins R, et al. Implementing a training intervention to support caregivers after stroke: a process evaluation examining the initiation and embedding of programme change. *Implementation Science* 2013;8(1):96. doi: 10.1186/1748-5908-8-96

25. Robson C. Real World Research,. Oxford: Blackwells 1995.

26. Edbrooke-Childs J, Hayes J, Sharples E, et al. Development of the Huddle Observation Tool for structured case management discussions to improve situation awareness on inpatient clinical wards. *BMJ quality & safety* 2018;27(5):365-72. doi: 10.1136/bmjqs-2017-006513 [published Online First: 2017/09/21]

27. Braun V, Clarke V. Using thematic analysis in psychology. *Qualitative Research in Psychology* 2006;3(2):77-101. doi: 10.1191/1478088706qp063oa

28. Gove D, Diaz-Ponce A, Georges J, et al. Alzheimer Europe's position on involving people with dementia in research through PPI (patient and public involvement). *Aging Ment Health* 2018;22(6):723-29. doi: 10.1080/13607863.2017.1317334 [published Online First: 2017/05/18]

29. Swarbrick CM, Doors O, Educate, et al. Visioning change: Co-producing a model of involvement and engagement in research (Innovative Practice). *Dementia (London, England)* 2016 doi: 10.1177/1471301216674559 [published Online First: 2016/10/19]

30. Morbey H, Harding AJE, Swarbrick C, et al. Involving people living with dementia in research: an accessible modified Delphi survey for core outcome set development. *Trials* 2019;20(1):12. doi: 10.1186/s13063-018-3069-6

For peer review only

Acknowledgements

The support of the Economic and Social Research Council (ESRC) and National Institute for Health Research (NIHR) is gratefully acknowledged. Work Programme 5, Developing the evidence base for evaluating dementia training in NHS hospitals (DEMTRAIN), forms part of the ESRC/ NIHR Neighbourhoods and Dementia mixed-methods Study (www.neighbourhoodsanddementia.org) under grant: ES/L001772/1. Our special thanks to Garstang Memory Café, Scottish Dementia Working Group, all of our co-researchers who have helped us, and continue to be an integral part of the DEMTRAIN study. We thank the participants and all who helped us in the study, especially the DEMTRAIN Advisory Group, Dr Carol Opdebeeck, Manchester Metropolitan University, and Marie Crane, Research Co-ordinator.

Authors' contributions:

SR, LD, DR and JK designed the DEMTRAIN study outline submitted to NIHR/ESRC. FA is the lead researcher on the study and led on developing the protocol for this publication. AH and HM are current researchers on the study. SR is the principal investigator for this study. JK is the chief investigator for all eight of the Neighbourhoods and Dementia study work programmes. DR, CS, LD, MH, FH, RE, IL, SB, and AB are part of the wider team and provide wider expertise. All authors contributed to the development of the study protocol and this publication.

Funding Statement

The support of the Economic and Social Research Council (ESRC) and National Institute for Health Research (NIHR). Work Programme 5, Developing the evidence base for evaluating dementia training in NHS hospitals (DEMTRAIN), forms part of the ESRC/ NIHR Neighbourhoods and Dementia mixed-methods Study (www.neighbourhoodsanddementia.org) under grant: ES/L001772/1.

Competing interests statement:

None

Word count: **4595**

For peer review only

Table 1: Case Study site selection considerations and sample matrix

<table>
<tr><th rowspan="2">Hospital</th><th rowspan="2">NAD Score*</th><th rowspan="2">CQC Scores**</th><th rowspan="2">Score discrepancy (NAD vs CQC)</th><th colspan="2">Patient Experience</th><th>Staff Experience</th><th rowspan="2">Dementia Specialist Ward in hospital</th><th rowspan="2">Dementia Lead present at either ward or directorate level</th><th rowspan="2">Geographic region
(i.e. Sustainability and Transformation Plans/STPs region)</th></tr>
<tr><th>Friends and Family Test score:

Inpatient</th><th>Friends and Family test score:

Outpatients</th><th>Friends and Family Test score:

Staff - Work</th></tr>
<tr><td>Site 1</td><td rowspan="6">Including a range of scores:

- High
- Mixed
- Low</td><td rowspan="6">Including a range of scores:

-Outstanding
-Good
-Requires improvement
-Inadequate</td><td rowspan="6">We aim to recruit a range of hospitals where there is ‘score discrepancy’ and where there is ‘no score discrepancy’ between the NAD and CQC scores.

Example 1 (discrepancy): NAD Score is High, yet CQC score is Inadequate.

Example 2 (no discrepancy): NAD Score is Low, and CQC score is Inadequate.</td><td colspan="3" rowspan="6">Mix of High and low scores: ranging between 50 - 100%</td><td rowspan="6">Yes or No</td><td rowspan="6">Yes or No</td><td rowspan="6">For example:

- North
- Midlands and East
- South West</td></tr>
<tr><td>Site 2</td></tr>
<tr><td>Site 3</td></tr>
<tr><td>Site 4</td></tr>
<tr><td>Site 5</td></tr>
<tr><td>Site 6</td></tr>
</table>

*NAD: National Audit of Dementia: The scores are based on 24 hospitals randomly sampled from stratified lists of hospitals scoring low, mixed and high across three main domains of dementia care (governance, staff training and patient care) derived from the NAD in 2016 for our staff survey sample selection.

**CQC: Care Quality Commission: The Score will be based on the Hospitals overall CQC score report: https://www.cqc.org.uk/

For peer review only - http://bmjopen.bmj.com/site/about/guidelines.xhtml

Figure 1: Recruitment process for acute NHS hospitals taking part in the DEMTRAIN Hospital Organisational Survey of dementia training and care in England

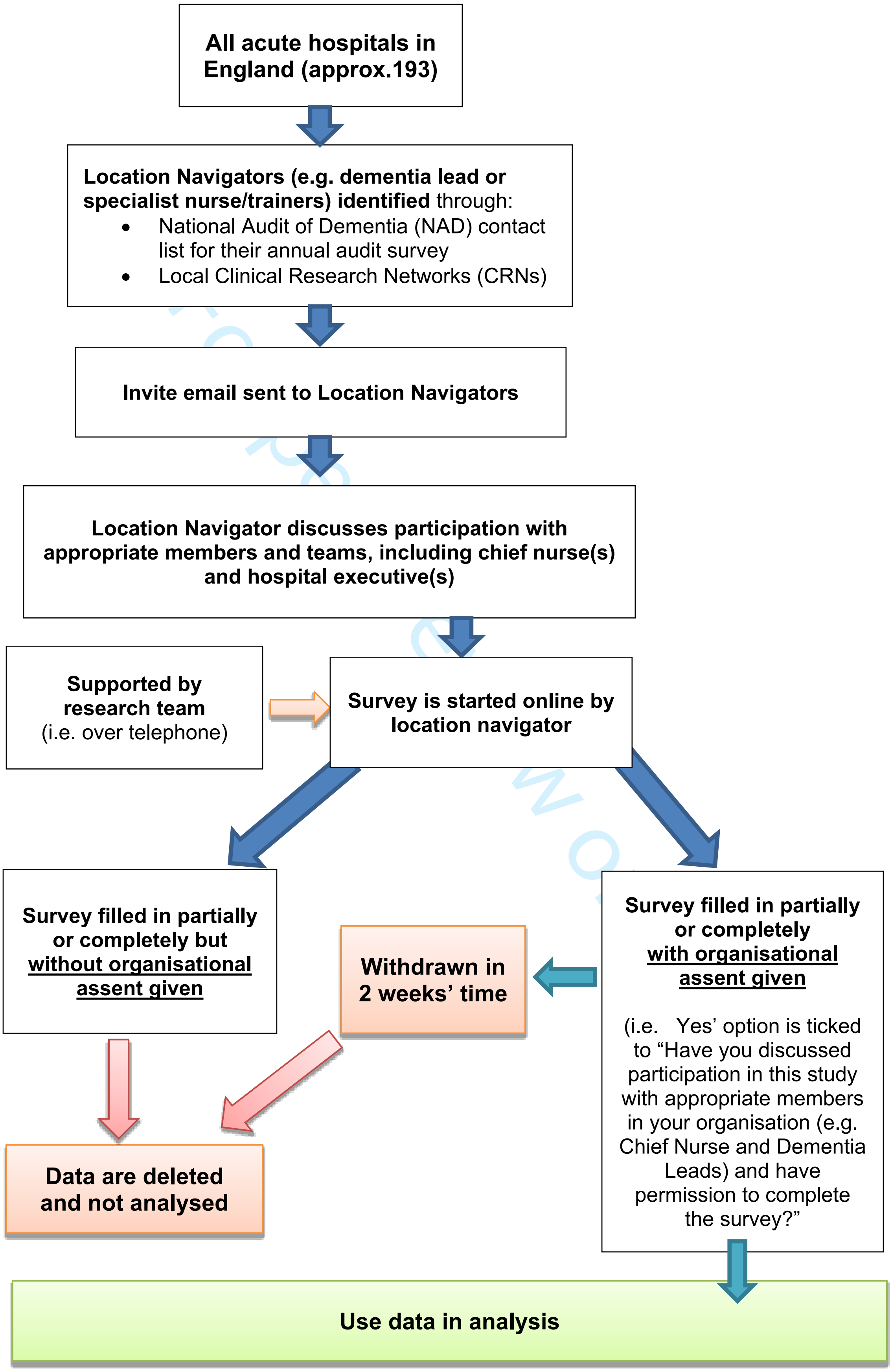

Figure 2: Proposed data collection structure in each of the six hospitals

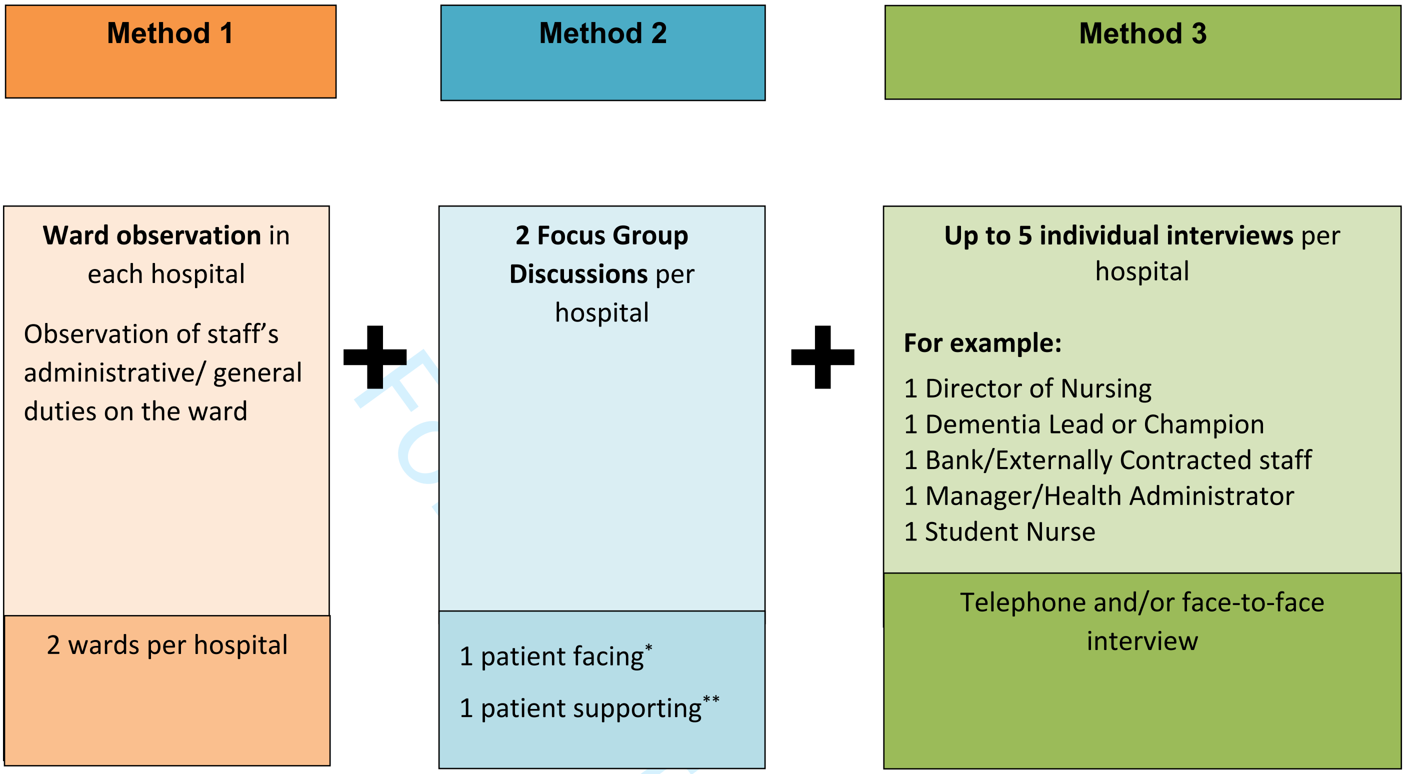

*Patient facing: Doctors, nurses, health care assistants, allied health professionals, including outpatient, A&E, X-rays, dietitians, frailty ward staff, student nurses.

**Patient supporting: Social care, voluntary (community and hospital), discharge staff, patient advice and liaison service staff, car park, & security staff, porters, and other domestic and support staffs

For peer review only

SPIRIT 2013 Checklist: Recommended items to address in a clinical trial protocol and related documents*

Section/item	Item No	Description	Addressed on page number
**Administrative information**			
Title	1	Descriptive title identifying the study design, population, interventions, and, if applicable, trial acronym	4
Trial registration	2a	Trial identifier and registry name. If not yet registered, name of intended registry	NA (page 5 Prospero reg)
	2b	All items from the World Health Organization Trial Registration Data Set	NA
Protocol version	3	Date and version identifier	NA
Funding	4	Sources and types of financial, material, and other support	17
Roles and responsibilities	5a	Names, affiliations, and roles of protocol contributors	17
	5b	Name and contact information for the trial sponsor	17
	5c	Role of study sponsor and funders, if any, in study design; collection, management, analysis, and interpretation of data; writing of the report; and the decision to submit the report for publication, including whether they will have ultimate authority over any of these activities	17
	5d	Composition, roles, and responsibilities of the coordinating centre, steering committee, endpoint adjudication committee, data management team, and other individuals or groups overseeing the trial, if applicable (see Item 21a for data monitoring committee)	17

For peer review only - http://bmjopen.bmj.com/site/about/guidelines.xhtml

**Introduction**			
Background and rationale	6a	Description of research question and justification for undertaking the trial, including summary of relevant studies (published and unpublished) examining benefits and harms for each intervention	4
	6b	Explanation for choice of comparators	NA
Objectives	7	Specific objectives or hypotheses	5, 7 and 8
Trial design	8	Description of trial design including type of trial (eg, parallel group, crossover, factorial, single group), allocation ratio, and framework (eg, superiority, equivalence, noninferiority, exploratory)	5, 7 and 8
**Methods: Participants, interventions, and outcomes**			
Study setting	9	Description of study settings (eg, community clinic, academic hospital) and list of countries where data will be collected. Reference to where list of study sites can be obtained	5-10
Eligibility criteria	10	Inclusion and exclusion criteria for participants. If applicable, eligibility criteria for study centres and individuals who will perform the interventions (eg, surgeons, psychotherapists)	5-10
Interventions	11a	Interventions for each group with sufficient detail to allow replication, including how and when they will be administered	NA
	11b	Criteria for discontinuing or modifying allocated interventions for a given trial participant (eg, drug dose change in response to harms, participant request, or improving/worsening disease)	NA
	11c	Strategies to improve adherence to intervention protocols, and any procedures for monitoring adherence (eg, drug tablet return, laboratory tests)	NA
	11d	Relevant concomitant care and interventions that are permitted or prohibited during the trial	NA
Outcomes	12	Primary, secondary, and other outcomes, including the specific measurement variable (eg, systolic blood pressure), analysis metric (eg, change from baseline, final value, time to event), method of aggregation (eg, median, proportion), and time point for each outcome. Explanation of the clinical relevance of chosen efficacy and harm outcomes is strongly recommended	NA
Participant timeline	13	Time schedule of enrolment, interventions (including any run-ins and washouts), assessments, and visits for participants. A schematic diagram is highly recommended (see Figure)	NA

For peer review only - http://bmjopen.bmj.com/site/about/guidelines.xhtml

Sample size	14	Estimated number of participants needed to achieve study objectives and how it was determined, including clinical and statistical assumptions supporting any sample size calculations	5-10
Recruitment	15	Strategies for achieving adequate participant enrolment to reach target sample size	5-7, and 12-13
**Methods: Assignment of interventions (for controlled trials)**			
Allocation:			
Sequence generation	16a	Method of generating the allocation sequence (eg, computer-generated random numbers), and list of any factors for stratification. To reduce predictability of a random sequence, details of any planned restriction (eg, blocking) should be provided in a separate document that is unavailable to those who enrol participants or assign interventions	NA
Allocation concealment mechanism	16b	Mechanism of implementing the allocation sequence (eg, central telephone; sequentially numbered, opaque, sealed envelopes), describing any steps to conceal the sequence until interventions are assigned	NA
Implementation	16c	Who will generate the allocation sequence, who will enrol participants, and who will assign participants to interventions	5-10
Blinding (masking)	17a	Who will be blinded after assignment to interventions (eg, trial participants, care providers, outcome assessors, data analysts), and how	NA
	17b	If blinded, circumstances under which unblinding is permissible, and procedure for revealing a participant's allocated intervention during the trial	NA
**Methods: Data collection, management, and analysis**			
Data collection methods	18a	Plans for assessment and collection of outcome, baseline, and other trial data, including any related processes to promote data quality (eg, duplicate measurements, training of assessors) and a description of study instruments (eg, questionnaires, laboratory tests) along with their reliability and validity, if known. Reference to where data collection forms can be found, if not in the protocol	NA
	18b	Plans to promote participant retention and complete follow-up, including list of any outcome data to be collected for participants who discontinue or deviate from intervention protocols	5-7, and 12-13

Data management	19	Plans for data entry, coding, security, and storage, including any related processes to promote data quality (eg, double data entry; range checks for data values). Reference to where details of data management procedures can be found, if not in the protocol	5-6 and 12-13
Statistical methods	20a	Statistical methods for analysing primary and secondary outcomes. Reference to where other details of the statistical analysis plan can be found, if not in the protocol	7-8, and 10-11
	20b	Methods for any additional analyses (eg, subgroup and adjusted analyses)	NA
	20c	Definition of analysis population relating to protocol non-adherence (eg, as randomised analysis), and any statistical methods to handle missing data (eg, multiple imputation)	NA
**Methods: Monitoring**			
Data monitoring	21a	Composition of data monitoring committee (DMC); summary of its role and reporting structure; statement of whether it is independent from the sponsor and competing interests; and reference to where further details about its charter can be found, if not in the protocol. Alternatively, an explanation of why a DMC is not needed	NA
	21b	Description of any interim analyses and stopping guidelines, including who will have access to these interim results and make the final decision to terminate the trial	NA
Harms	22	Plans for collecting, assessing, reporting, and managing solicited and spontaneously reported adverse events and other unintended effects of trial interventions or trial conduct	NA
Auditing	23	Frequency and procedures for auditing trial conduct, if any, and whether the process will be independent from investigators and the sponsor	NA
**Ethics and dissemination**			
Research ethics approval	24	Plans for seeking research ethics committee/institutional review board (REC/IRB) approval	12
Protocol amendments	25	Plans for communicating important protocol modifications (eg, changes to eligibility criteria, outcomes, analyses) to relevant parties (eg, investigators, REC/IRBs, trial participants, trial registries, journals, regulators)	12-13

For peer review only - http://bmjopen.bmj.com/site/about/guidelines.xhtml

Consent or assent	26a	Who will obtain informed consent or assent from potential trial participants or authorised surrogates, and how (see Item 32)	5-10
	26b	Additional consent provisions for collection and use of participant data and biological specimens in ancillary studies, if applicable	NA
Confidentiality	27	How personal information about potential and enrolled participants will be collected, shared, and maintained in order to protect confidentiality before, during, and after the trial	12-13
Declaration of interests	28	Financial and other competing interests for principal investigators for the overall trial and each study site	NA
Access to data	29	Statement of who will have access to the final trial dataset, and disclosure of contractual agreements that limit such access for investigators	NA
Ancillary and post-trial care	30	Provisions, if any, for ancillary and post-trial care, and for compensation to those who suffer harm from trial participation	NA
Dissemination policy	31a	Plans for investigators and sponsor to communicate trial results to participants, healthcare professionals, the public, and other relevant groups (eg, via publication, reporting in results databases, or other data sharing arrangements), including any publication restrictions	2, 12, 14
	31b	Authorship eligibility guidelines and any intended use of professional writers	17
	31c	Plans, if any, for granting public access to the full protocol, participant-level dataset, and statistical code	NA
**Appendices**			
Informed consent materials	32	Model consent form and other related documentation given to participants and authorised surrogates	NA
Biological specimens	33	Plans for collection, laboratory evaluation, and storage of biological specimens for genetic or molecular analysis in the current trial and for future use in ancillary studies, if applicable	NA

*It is strongly recommended that this checklist be read in conjunction with the SPIRIT 2013 Explanation & Elaboration for important clarification on the items. Amendments to the protocol should be tracked and dated. The SPIRIT checklist is copyrighted by the SPIRIT Group under the Creative Commons "Attribution-NonCommercial-NoDerivs 3.0 Unported" license.

BMJ Open

Developing the evidence base for evaluating dementia training in NHS hospitals (DEMTRAIN): a mixed methods study protocol

Journal:	*BMJ Open*
Manuscript ID	bmjopen-2019-030739.R1
Article Type:	Protocol
Date Submitted by the Author:	19-Sep-2019
Complete List of Authors:	Ahmed, Faraz; Lancaster University Faculty of Health and Medicine, Health Research Division<br>Morbey, Hazel; Lancaster University Faculty of Health and Medicine, Health Research Division<br>Harding, Andrew; Lancaster University, Division of Health Research, Faculty of Health and Medicine<br>Reeves, David; The University of Manchester, Division of Population Health, Health Services Research & Primary Care<br>Swarbrick, Caroline; Lancaster University Faculty of Health and Medicine, Health Research Division<br>Davies, Linda; University of Manchester, Division of Population Health, Health Services Research & Primary Care<br>Hann , Mark ; University of Manchester, Division of Population Health, Health Services Research & Primary Care<br>Holland, Fiona; University of Manchester, Division of Population Health, Health Services Research & Primary Care<br>Elvish, Ruth; University of Manchester, Division of Nursing, Midwifery & Social Work<br>Leroi, Iracema; University of Manchester, Division of Neuroscience & Experimental Psychology<br>Burrow, Simon; University of Manchester, Division of Nursing, Midwifery & Social Work<br>Burns, Alistair; University of Manchester, Division of Neuroscience & Experimental Psychology<br>Keady, John; University of Manchester, Division of Nursing, Midwifery & Social Work<br>Reilly, Siobhan; Lancaster University, Division of Health Research
**Primary Subject Heading**:	Health services research
Secondary Subject Heading:	Qualitative research, Health informatics, Health economics, Epidemiology
Keywords:	Dementia < NEUROLOGY, Hospitals, Dementia training, Mixed methods, EDUCATION & TRAINING (see Medical Education & Training)

SCHOLARONE™
Manuscripts

Title: Developing the evidence base for evaluating dementia training in NHS hospitals (DEMTRAIN): a mixed methods study protocol

Authors:

Faraz Ahmed [a]
Hazel Morbey [a]
Andrew Harding [a]
David Reeves [b]
Caroline Swarbrick [a]
Linda Davies [b]
Mark Hann [b]
Fiona Holland [b]
Ruth Elvish [c]
Iracema Leroi [d]
Simon Burrow [c]
Alistair Burns [d]
John Keady [c]
Siobhan T. Reilly [a]

[a] Lancaster University, Division of Health Research, Lancaster University, Lancaster LA1 4YG

[b] Division of Population Health, Health Services Research & Primary Care, University of Manchester, Jean McFarlane Building, Oxford Road, Manchester M13 9PL

[c] Division of Nursing, Midwifery & Social Work, University of Manchester, Manchester, Oxford Road, Manchester M13 9PL

[d] Division of Neuroscience & Experimental Psychology, University of Manchester, Manchester, Oxford Road, Manchester M13 9PL

Corresponding Author:

Siobhan T. Reilly, Lancaster University, Division of Health Research, Lancaster University, Lancaster LA1 4YG **Email:** s.reilly@lancaster.ac.uk **Tel:** + 44(0)1524594367

For peer review only

Abstract:

Introduction: Around 70 percent of acute hospital beds in the UK are occupied by older people, approximately 40 percent of whom have dementia. Improving the quality of care in hospitals is a key priority within national dementia strategies. Limited research has been conducted to evaluate dementia training packages for staff, and evaluation of training often focuses on immediate, on-the-day training feedback and effects.

Objectives: Our study aims to answer two research questions:

(1) How do variations in content, implementation and intensity of staff dementia training in acute hospitals in England relate to health service outcome/process measures and staff outcomes?

(2) What components of staff dementia training are most strongly related to improved patient and staff outcomes?

Methods and analysis: Using the principles of programme theory, a mixed method study will be used to identify mechanisms and the interactions between them, as well as facilitators and barriers to dementia training in hospitals. We will use existing data, such as Hospital Episode Statistics, alongside two surveys (at hospital and staff level).We will recruit up to 193 acute hospitals in England to participate in the hospital level survey. We aim to recruit up to 30 staff members per hospital, from a random sample of 24 hospitals. In addition, we will explore the cost effectiveness of dementia training packages and carry out an in-depth case study of up to six hospitals.

Ethics and dissemination: The study has been reviewed and approved by the Faculty of Health and Medicine Research Ethics Committee (FHMREC 17056) and HRA (IRAS ID 242166: REC reference 18/HRA/1198). We plan to develop both standard (e.g. academic publications, presentations at conferences) and innovative (e.g. Citizen Scientist web portals, online fora, links with hospitals and third sector organisations) means of ensuring the study findings are accessible and disseminated regionally, nationally and internationally.

For peer review only

For peer review only - http://bmjopen.bmj.com/site/about/guidelines.xhtml

Article Summary

Strengths and limitations of this study

- This study will use a range of existing datasets (including Hospital Episode Statistics, and National Audit of Dementia's Organisational Checklist) and mixed methods approaches to make recommendations about dementia training in acute hospital settings.

- Using the principles of programme theory, we will expand on mechanisms and the interactions between them, as well as facilitators and barriers to dementia training in the hospitals.

- This study aims to explore the long term impact of dementia training (if any) on health service outcomes/ process measures (e.g. length of stay, emergency re-admissions and mortality) and staff outcomes (e.g. confidence, staff strain, knowledge of dementia).

- Given the multiple initiatives and dementia policies employed in hospitals across England, it will be difficult to establish the effects of dementia training directly on patient and staff outcomes.

Keywords:

Dementia, Hospitals, Dementia training, Mixed methods

For peer review only - http://bmjopen.bmj.com/site/about/guidelines.xhtml

Introduction

Improving the quality of care in general hospitals continues to be one of the key priorities within national dementia strategies [1-3]. Acute NHS Trusts provide a range of services, including accident and emergency departments, inpatient and outpatient medicine and surgery and in some cases very specialist medical care [4]. They provide secondary care, ranging from relatively small district hospitals to large city teaching hospitals in England. The term acute, generally refers to physical illnesses and conditions, which are usually short-term and require diagnostic tests, treatment and follow-up care [4 5]. At any one time, around 70 percent of acute hospital beds are occupied by older people, 40% of whom have dementia [6-8]. Despite limited literature in the area, a recent ethnography of the care received by people living with dementia in acute hospital suggest that staff struggle to respond to the needs of people living with dementia in acute care settings and that training may be one of the intervention that can help to address quality of care issues observed [9]. Dementia awareness and care training is widely thought to increase staff competencies [10 11], although types of training and how effectiveness is assessed is highly variable.

Since 2013, Health Education England (HEE) has overseen increases in the provision of foundational-level dementia training [12 13]. However, limited research has so far been conducted to evaluate dementia training packages. Evaluation tends to focus on immediate, on-the-day training feedback and effects. Moreover, training for staff in any care setting is seldom evaluated for its impact on the care of people living with dementia and for long term effects and impact on staff [14 15]. Given the high variability of dementia training packages [11], there is a need to better understand the current evidence and context of dementia training that is provided in acute NHS hospitals. This study will explore the complex workings of dementia training, including exploring relationships with patient and staff outcomes. This mixed methods study aims to address two overarching research questions:

1) How do variations in content, implementation and intensity of dementia training in hospitals in England relate to health service outcome/process measures (e.g. length of stay, emergency re-admissions and mortality) and staff outcomes (e.g. confidence, staff strain, knowledge of dementia)?
2) What components of dementia training are most strongly related to improved patient and staff outcomes?

The findings will lead to a set of specific recommendations and guidance about which elements of training packages are more effective and appropriate for use in acute hospitals.

Methods and analysis

The three phases of the study are:

- **Phase 1** Mapping the evidence base for dementia training in hospitals
- **Phase 2** Cohort study to assess differences in acute NHS hospital outcomes for people living with dementia compared to those without, and the impact of training methods on patient outcome.

For peer review only - http://bmjopen.bmj.com/site/about/guidelines.xhtml

- **Phase 3** Multiple hospital case studies of staff dementia training and care in acute NHS hospitals.

Our mixed methods study will utilise the principles of programme theory [16] to identify mechanisms and the interactions between them, as well as the facilitators and barriers to dementia training in the hospital. DEMTRAIN, one of eight work programmes within the Neighbourhoods and Dementia Study (funded by the ESRC/NIHR), is developing the evidence base for evaluating dementia training in acute NHS hospitals. The DEMTRAIN study aims to integrate the various phases, which includes quantitative and qualitative data (see figure 1).

INSERT FIGURE 1

Phase 1: Mapping the evidence base for dementia training in Hospitals

Aims:

1. To review the current evidence base for staff dementia training in hospital settings
2. To map the variation in staff dementia training currently provided to acute hospital staff in England
3. To assess the impact of differences in staff dementia training upon staff knowledge, practice, organisational culture and staff strain in acute NHS hospitals.

Methods:

Phase 1 will involve three main elements:

I. We will conduct **a systematic review** of the evidence base for training hospital staff in dementia awareness and care exploring both the quality and effectiveness of training programmes. This will be achieved by conducting a critical interpretive synthesis [17], which will form the basis of a logic model using the principles of programme theory to identify mechanisms, interaction, facilitators and barriers to dementia training in hospital settings. The protocol for this element of phase 1 is registered on PROSPERO and is not outlined here [18].

II. We will conduct an **organisational surveys** in acute NHS hospitals in England (approx. 193 hospitals will be approached using CCQI National Audit of Dementia (NAD) sample frame contact list) to identify training strategies; establish the presence, frequency, duration, characteristics and format of training programmes; quality of training and resources utilised in developing training. We will also explore what other initiatives are implemented (e.g. having a dementia champion, active participation in Butterfly scheme, Dementia Friends, carers allowed on wards at meal times).

III. We will conduct a survey of staff (**hospital staff survey**) to collect data on the extent of training, knowledge, attitudes, staff strain, organisational culture and satisfaction in caring for patients living with dementia. We aim to recruit 24 hospitals randomly

For peer review only - http://bmjopen.bmj.com/site/about/guidelines.xhtml

sampled from stratified lists of hospitals scoring low, mixed and high across three main domains of dementia care (governance, staff training and patient care) derived from the NAD in 2016).

Recruitment for organisational survey in acute NHS hospitals in England

We will use NAD data to identify potential hospitals for the hospital organisational survey. Our preliminary discussions with our advisory group (including representatives from NAD who are involved in similar surveys, and local Dementia Leads who are hospital staff responsible for ensuring that their hospital is aligned with NHS Trust and National dementia strategies, and involved in developing and implemented dementia training in their hospitals), suggests that in most cases Dementia Leads at each hospital will have access to the data needed to complete the organisational level survey. However, given the segmented and large variations in recording systems for dementia training across Hospitals in England, the information needed to complete the survey may be held in other departments in the hospital, such as other IT or human resources (HR) systems. On this basis, we will have multiple survey collection approaches (i.e. online and telephone completion), with researcher-led support to ensure high quality data and completion. Figure 2 presents the hospital recruitment process for the Organisational Survey.

INSERT FIGURE 2

Dementia Leads will be responsible for completing the data. However, in cases where the information needed to complete the Hospital Organisational Survey is not directly available to the Dementia Leads, the Dementia Leads will act as Location Navigators and will direct requests to complete the survey to the appropriate person in the acute NHS hospital.

Before starting the organisational survey, participants will be asked if they have discussed participation in this study with appropriate members in their organisation, and that their hospital and or Trust has given assent to participate; where assent is not given, survey data will be deleted and not analysed.

Recruitment for staff survey in two specific wards in acute NHS hospitals in England

Using the NAD returns for 2016, we will combine across sets of related question items to construct performance scores for each hospital on three main domains of care for inpatients with dementia: governance; staff training; and patient care (policies/processes concerning nutrition, discharge, assessment, communication and carers' rating of patient care). We will then identify subgroups of hospitals scoring high, low and mixed across the three domains. Within each of these subgroups hospitals we will randomly sample for invitation into the survey, with the objective of recruiting 8 hospitals from within each subgroup (24 in total).

For peer review only

For peer review only - http://bmjopen.bmj.com/site/about/guidelines.xhtml

We will survey different types of staff members working on two contrasting acute wards where dementia is expected to be prevalent:

- **Ward 1: Elderly Care /Older people's ward** (if the acute NHS hospital does not have these wards, an **orthopaedic ward** will be selected instead)

- **Ward 2: General Surgery or surgical ward** (if the acute NHS hospital does not have these wards, an **oncology ward** will be selected instead)

We will recruit staff from five categories in each acute NHS hospital:

1) Doctors
2) Nurses
3) Health Care Assistants
4) Allied healthcare professionals (e.g. physiotherapists, dieticians)
5) Support staff in the hospital (e.g. housekeepers, porters, receptionists)

In view of our member involvement (alternatively termed as Patient and Public Involvement) and Advisory Group feedback, our sample will focus on staff categories with more frequent interactions with patients living with dementia. It is thought that the above staff groups may be more likely to affect patient-related processes of care and outcomes. Staff will be sent electronic or postal copies of the staff survey depending on individual preference.

Location Navigators and local Clinical Research Networks (CRNs) will help identify 15-30 participants from each hospital. The research team will inform location navigators and CRN to recruit staff in a particular ward on a particular day and to select a random sample from ward/hospital staff lists/registers (e.g. every fourth staff member on the list will be approached). However, this may not be possible in the absence of staff lists/registers. Data on representativeness of the sample in relation to the ward will also be collected (i.e. estimated number of staff on ward on a given day, in comparison to the number of surveys completed).

Phase 2: Cohort study to assess differences in acute NHS hospital outcomes for people living with dementia compared to those without, and the impact of training methods on patient outcome

Aim:

To determine the types and elements of training that are most strongly associated with hospital outcomes for people living with dementia, including length of stay, emergency readmissions (ERs within 30 days of prior discharge), and mortality (death within 30 days of discharge) for older people.

For peer review only - http://bmjopen.bmj.com/site/about/guidelines.xhtml

Methods:

The primary source of information on hospital outcomes will be the Hospital Episode Statistics (HES) database, which holds routinely collected information on all inpatient spells in England and Wales. Outcomes for patients with a diagnosis of dementia will be compared to outcomes for patients without a diagnosis, with a focus on how hospital training scores relate to the difference in outcomes between these two groups. Comparisons will be made separately for the financial years 2010/11, 2012/13 and 2016/17 to coincide with data on staff training and other dementia initiatives in hospitals collected under the NAD (see below). Patients living with dementia will be defined as those with a diagnosis of dementia reported in the hospital record at any time over the previous 5 years; patients without a diagnosis will be used as controls. This approach has been used in several previous studies (e.g. Care Quality Commission, State of health care and social care in England in 2012/13 Technical Annex 3 [19]) and the rate of misclassification is believed to be acceptably small [20]. Corresponding NAD data for 2010/11, 2012/13 and 2016/17 will be used to construct hospital-level scores on a number of key domains of dementia care, in particular governance, staff training and patient care.

Details of additional secondary research data

We will use other relevant secondary data where appropriate. In addition, we will develop a number of collaborations and explore other sources of data, such as data already available in the public domain or routinely collected as part of service evaluation. These may include further information on care, details and rates of staff training and patient experience among people living with dementia.

Phase 3: Multiple hospital case studies of staff dementia training and care in acute NHS hospitals

Aims:

1) To determine the content, implementation and intensity of training in contrasting hospitals and to explore the acceptability and feasibility of training to a range of hospital staff.

2) To identify barriers and facilitators for the implementation, and sustainability of staff dementia training in acute NHS hospitals.

3) To identify lessons about the implementation of staff dementia training that might be applicable in other care settings.

Methods:

Case study approaches in health research tend to involve multiple methods and offer pragmatic and appropriate approaches to provide an in-depth and strong focus on being able to report on how findings are contingent on context [21 22]. Observational methods further enhance the possibility for insights in case study research [23]. Our design includes multiple

For peer review only - http://bmjopen.bmj.com/site/about/guidelines.xhtml

data collection approaches, including observations, focus group and interviews within each case study hospital. This phase of the study is predominately qualitative with data collection from multiple sources with a range of stakeholders within each hospital. We will utilise the principles of programme theory and realist approach to identify theories of change with respect to training initiatives in different hospital contexts. For instance to identify mechanisms and the interactions between them to explain what has caused an outcome and in what context [24 25], as well as facilitators and barriers to dementia training in the hospital and its related outcomes [24]. Programme theory, within the theory-driven evaluation field, refers to a variety of ways of describing and evaluating a programme or intervention (such as staff dementia training) by developing an understanding of the causal modal that connect the inputs and activities of an intervention to its outcomes [16].

Using an organizational case study design we will explore the implementation of training practices in hospitals [26]. Using data from Phase 1, we will select up to six contrasting hospitals based on a number of factors related to the implementation of training such as: intensity, duration coverage of training; geography (Sustainability and Transformation Plans region and hospital type) (see table 1). A series of semi-structured interviews and focus groups will be conducted with a purposive sampling strategy to recruit staff with different levels of training and experience (including, doctors, nurses, support and domestic staff). These interviews and focus groups will explore expectations and experiences of staff dementia training among hospital staff. In addition, researchers will observe staff activity on wards at up to two shift changeovers. Observations will focus on administrative/general duties and the work environment related to dementia care. Researchers will visit case study hospitals over three days, guided by the Edbrooke-Childs *et al*. (2018) Huddle Observation Tool for inpatient clinical wards (please see below for more details on observations of staff on ward).

For peer review only

For peer review only - http://bmjopen.bmj.com/site/about/guidelines.xhtml

Table 1: Case Study site selection considerations and sample matrix

Hospital	NAD Score*	CQC Scores**	Score discrepancy (NAD vs CQC)	**Patient Experience**		**Staff Experience**	**Dementia Specialist Ward in hospital**	**Dementia Lead present at either ward or directorate level**	**Geographic region** (i.e. Sustainability and Transformation Plans/STPs region)
				Friends and Family Test score: Inpatient	*Friends and Family test score: Outpatients*	*Friends and Family Test score: Staff - Work*			
Site 1	Including a range of scores:<br><br>- High<br>- Mixed<br>- Low	Including a range of scores:<br><br>-Outstanding<br>-Good<br>-Requires improvement<br>-Inadequate	We aim to recruit a range of hospitals where there is 'score discrepancy' and where there is 'no score discrepancy' between the NAD and CQC scores.<br><br>Example 1 (discrepancy): NAD Score is High, yet CQC score is Inadequate.<br><br>Example 2 (no discrepancy): NAD Score is Low, and CQC score is Inadequate.	Mix of High and low scores: ranging between 50 - 100%			Yes or No	Yes or No	For example:<br><br>- North<br>- Midlands and East<br>- South West
Site 2									
Site 3									
Site 4									
Site 5									
Site 6									

*NAD: National Audit of Dementia: The scores are based on 24 hospitals randomly sampled from stratified lists of hospitals scoring low, mixed and high across three main domains of dementia care (governance, staff training and patient care) derived from the NAD in 2016 for our staff survey sample selection.

**CQC: Care Quality Commission: The Score will be based on the Hospitals overall CQC score report: https://www.cqc.org.uk/

For peer review only - http://bmjopen.bmj.com/site/about/guidelines.xhtml

Recruitment and data collection in case study sites

Case study site visits will take place over a three-day period to undertake interviews, focus groups and observational data collection (for more detail see figure 3, and Table 1).

Insert Figure 3 here

Observations of staff on ward

We will observe staff activity on wards at up to two staff shift changeovers, focusing on their administrative/general duties and the work environment. For example, before the start of the shift, or observation of domestic staff and porters at meals times. The observations will be guided by the Edbrooke-Childs *et al*. [27] Huddle Observation Tool for inpatient clinical wards. This approach explores four main areas on the ward in relation to staff general/administrative duties:

1. 'Risk management' (e.g. 'Were there opportunities to identify risks and come up with concrete plans for these risks?')
2. 'Structure' (e.g. 'Did the activities have a clear structure?')
3. 'Collaborative culture' (e.g. 'Did everyone have the opportunity to contribute and were all points of view respected?')
4. 'Environment' (e.g. Physical and emotional environment)

The Huddle Observation Tool was developed as an observational assessment tool to assess the team processes occurring during huddles, including the effectiveness of hospital huddles to provide structured case management discussions to improve situation awareness on inpatient clinical wards [27]. It, therefore, provides a systematic manner to explore the interaction between staff within a hospital ward environment. Moreover, any relevant observations that do not fit the four areas (risk management, structure, collaborative culture and environment) of the Huddle Observation Tool, can be added as additional notes. Researchers will only make observation notes during a visit. Observations will not be connected to a specific individual or staff member. It will be made clear and staff will be reassured, that the aim of observation of administrative and general duties is to identify best practice rather than highlight faults. Permissions to conduct the observation on a ward will be sought from the Location Navigator (e.g. ward managers/Dementia Leads), except for in a public space (e.g. main entrance or reception area). All researchers will have appropriate approvals from local R&D and HRA, including relevant training (e.g. research passport and good clinical practice).

Interviews and focus groups of hospital staff

We will aim to conduct two focus groups per hospital (total: 12 groups) with a range of staff – Group one will be with 'patient facing staff' and Group two will be with 'patient supporting' staff. Location navigators and CRN will help identify staff and distribute information sheets and consent forms. Participants will be selected purposively to allow a maximum of 10 participants per focus group:

For peer review only - http://bmjopen.bmj.com/site/about/guidelines.xhtml
For peer review only

1. **Patient facing (Group 1):** Doctors, nurses, health care assistants, allied health professionals, including outpatient, A&E, X-rays, dietitians, frailty ward staff, student nurses.
2. **Patient supporting (Group 2):** Social Care, Voluntary (Community and Hospital), Discharge Staff, Patient Advice and Liaison Service staff, Car Park, & Security Staff, Porters, and other Domestic and support staff

Our preliminary discussions with our advisory group (including local Dementia Leads responsible for ensuring that their hospital is aligned with NHS Trust and National dementia strategies, and involved in developing and implemented dementia training in their hospitals), indicated that separating 'patient facing' staff from 'patient supporting' staff would facilitate greater discussion of dementia training. Basic dementia knowledge (due to clinical training) and access to training is likely to differ considerably between the two groups, which may mean that some non-clinical staff (e.g. domestic staff) may be hesitant to talk about their dementia knowledge and access to dementia training in front clinical staff.

We will conduct up to five interviews per hospital with a range of staff, including:

- Director of Nursing
- Dementia Lead or Champion
- Bank/Externally Contracted staff
- Manager/Health Administrator
- Student Nurse

We estimate each interview and focus group to last on average an hour and will be audio recorded on encrypted devices. The group will be shown a summary of their hospital's performance in seven areas, which is currently available from NAD. This will encourage greater engagement and interaction between the participants and serve to prompt discussion. We expect the focus groups to take place at the hospitals.

Analysis of data

Quantitative analysis

We will use Stata to analyse the quantitative data collected. The main focus will be to examine how hospital training scores relate to the difference in outcomes between patients with a known diagnosis of dementia, compared to outcomes for patients without a known diagnosis. We will develop a number of statistical models to determine the types and elements of training that are most strongly associated with changes in the LoS, emergency readmissions within 30 days of prior discharge, outcomes related to the care received in the hospital for people living with dementia and mortality (death within 30 days of discharge) for older people. We will use NAD data (2010/11, 2012/13 and 2016/17) to construct hospital-level scores on a number of key domains of dementia care, in particular governance, staff training and patient care. Statistical models will be fitted to assess relationships between scores on the different domains of dementia care and differences in outcomes between people living with dementia compared to those without, after controlling for a range of confounding variables including

For peer review only - http://bmjopen.bmj.com/site/about/guidelines.xhtml

patient demographics, pre-existing health and reasons for hospital admission. A further analysis will examine relationships between outcomes and dementia-care scores derived from our own hospital organisational survey in 2016/17 only.

Qualitative analysis

A thematic analysis [28] will be undertaken to identify emerging themes (including a-priori themes, in view of the literature and current dementia policies) in relation to case study interviews, focus groups and observations. Data will be imported, coded and managed using the qualitative data analysis software tool (NVivo v11). The analysis process will be guided by our programme theory that is being developed as part of a systematic review (i.e. critical interpretive synthesis) of the current evidence-base on dementia training in hospital settings [18].

Economic analysis

A combined decision analytic/simulation model will be developed from the Phase 1 and 3 data and review of the literature. The economic evaluation will use data from Phases 1-3 to develop the model structure and explore the costs, outcomes and relative cost effectiveness of alternative training interventions from the perspective of health and social care services and patients. The primary measure of benefit will be the Quality Adjusted Life Year (QALY) measure. Incremental cost-effectiveness ratios (ICERs) will be calculated. Cost-effectiveness acceptability curves, the probability of whether training is cost-effective, and net benefit statistics will be estimated to explore uncertainty in the data. One and multi-way sensitivity analyses will be used to explore the impact of alternative training methods over alternative time horizons (e.g. 5 and 10 years), patient populations and settings, alternative data sources and range of costs are included. This will include assessment of the impact of barriers and facilitators to implementing training and staff participation and compliance with training.

DEMTRAIN Patient and Public Involvement and Co-research model.

There is increasing recognition that inclusion of people living with dementia beyond the realm of 'participant' to involvement in all areas of the research process in ways which are both personally meaningful and relationally supportive is imperative [29]. The CO-research INvolvement and Engagement in Dementia or COINED model [30] forms part of the work of the Neighbourhoods and Dementia Study and is a unique and positive feature of the DEMTRAIN study. The term 'co-researcher' reflects collaborative, co-operative and community-based partnership between groups of people living with dementia, academic researchers and service providers. We have involved people living with dementia and their care partners in a range of areas including:

- development of the organisation and staff level survey,
- development of qualitative interview and focus group guide,
- regular feedback on study design and implementation.

Care partners' is the term selected by Co-research involvement groups of our Neighbourhoods Study, to refer to those who look after, support and care for someone living with dementia, in a non-professional, non-paid capacity [31]. This may be a family member, friend or neighbour.

For peer review only - http://bmjopen.bmj.com/site/about/guidelines.xhtml

The qualitative analysis of case study data provides an additional opportunity to bring in the perspectives of people living with dementia and their care partners at the analysis stage as co-researchers. We, therefore, propose various activities that will help in the interpretation of the data/findings and help us bring their valuable perspectives:

- Co-researchers will inform the development of the DEMTRAIN programme theory, that is part of a systematic review (i.e. critical interpretive synthesis) of the current evidence-base on dementia training in hospital settings, and that serves as theoretical framework to our study.
- Co-research observational visits to hospital public spaces (e.g. main reception and public eating areas), in up to six sites, with researchers who are involved in qualitative data collection for case studies.
- Co-researchers will review various anonymised iterations of the analysed summary/data.

All co-researchers will receive pre- and post-visit briefing before each hospital visit to discuss any issues or challenges concerning their observations. They will be accompanied by a researcher at all times and, if they wish, an informal care partner may escort them during the visit. Given the nature of the research and data collection, we envisage no risk to researchers. However, Work Programme 8 (WP8) provides a well-being service for all Programme staff, co-researchers and members working within the Neighbourhoods Study. WP8 is led by Dr Ruth Elvish, clinical psychologist, who provides this confidential support service.

Ethics and dissemination

The study has been reviewed and approved by the Faculty of Health and Medicine Research Ethics Committee (FHMREC 17056) and HRA (IRAS ID 242166: REC reference 18/HRA/1198). We plan to develop both standard (e.g. academic publications, presentations at conferences) and innovative (e.g. Citizen Scientist web portals, online fora, links with hospitals and third sector organisations) means of ensuring the study findings are accessible and disseminated regionally, nationally and internationally.

Ethical considerations

Given the nature of the topics and methods adopted, the study poses a low risk. However, we understand that hospital staff may be anxious or fearful of reprisal from other colleagues or hospital management when completing a survey, such as our hospital staff survey, that explores their skills, competencies and satisfaction in regards to dementia training (particularly if the training is developed and provided by their own hospital). To manage these risks all participant answers will be separate from any identifiable information, and we will adhere to Lancaster University guidelines on confidentiality and anonymity of participants. A key issue concerning staff observation is whether the individual concerned is ‘identifiable’ from the information collected (i.e. in the observation notes). However, we will exclude any identifiable information in the notes and focusing only on administrative/general duties and the work environment.

For peer review only

For peer review only - http://bmjopen.bmj.com/site/about/guidelines.xhtml

Information provided to staff invited to complete a survey will ensure they are aware of this anonymity and that they are under no obligation to take part in the study. Participants will also be made aware that they are free to withdraw from a survey/interview/focus group if they wish. Staff will be informed that once a survey/interview/focus group is complete they have two weeks to withdraw the survey by submitting a request to the research team. Consent to participate in the staff survey will be taken as granted on completion of the survey. Before each interview and focus group, a researcher will go through the study information sheet and seek informed consent for each participant. Staff will be made aware that they are free to leave an interview or focus group if they wish. The consent forms will ensure that staff are aware that their participation is confidential, and that we will be audio recording the discussions.

Any quotes or reporting will not identify individual staff or their hospital. Audio files will be stored safely and securely in line with university and our study data management plan (DMP). For instance, all digital data (i.e. audio recordings) will be stored on encrypted audio recorders (and backed up at the earliest opportunity onto encrypted hard drives and subsequently onto secure University servers. Only the research team and those involved in transcribing the data will have access to the raw data. All participant information will be stored in locked cabinets.

All staff participating in the study (i.e. staff survey, Interview and focus group participants) will be offered a £10 voucher as acknowledgement of their contribution and time. Similarly, CPD certificates for participation in research will be provided to all staff members who indicate that they wish to receive one. As thanks for participation in the study, we will offer a Dementia Clock to the hospital wards taking part in the staff survey (up to two Dementia Clocks per hospital). Summary reports based on the primary data collection and secondary data (i.e. HES and NAD data) will be made available to all hospitals who complete the hospital organisational survey and indicate that they wish to receive one. Given individual staff responses will be confidential this summary will not include information relating to the staff survey. Completed organisational surveys will be entered into a prize draw for up to 10 hospitals to receive £500 for either equipment that enhances the hospital experience for patients living with dementia or a dementia-related charitable donation.

Strengths and limitations

This study will use the principles of programme theory to expand on mechanisms and the interactions between them, facilitators and barriers to dementia training in acute hospitals in England. One of the key strengths of this study is that it uses a range of existing datasets (including Hospital Episode Statistics and National Audit of Dementia and mixed methods approaches to make recommendations in relation to dementia training in acute hospital settings. Unlike previous studies that have focused on immediate, on-the-day training feedback and effects of dementia training in hospital settings [10 11], our proposed study will attempt to explore the longer-term impact of dementia training. Given the multiple initiatives and dementia policies employed in hospitals across England, it will be challenging to establish the effects of dementia training directly on patient and staff outcomes. Nonetheless, we will use statistical models and mixed methods approaches to adjust and triangulate our findings.

For peer review only

Another key strength of our study is that across each of the study phases, we will continue to facilitate the involvement of people living with dementia as co-researchers, guided by the COINED model of co-research. As well as, consultations with our collaborators and key stakeholders involved in similar form of studies, such as What Works' in Dementia Education and Training evaluation (http://www.leedsbeckett.ac.uk/pages/what-works/), National Audit of Dementia (NAD), and local Clinical Research Network (CRN). Our project partner, Advancing Quality Alliance (AQuA) an improvement body working across the NHS in the North West will also be consulted to aide recruitment and dissemination of the findings.

References

1. Department of Health. Prime Minister's challenge on dementia 2020. London, 2015.

2. Alzheimer's Society. Building dementia-friendly communities: A priority for everyone, 2013.

3. Department of Health. Living well with dementia. A national dementia strategy, 2009. London: Department of Health 2009.

4. NHS. NHS authorities and trusts 2016 [Available from: https://www.nhs.uk/using-the-nhs/about-the-nhs/nhs-authorities-and-trusts/ accessed 6 March 2019.

5. Northern Devon Healthcare NHS Trust. Definitions 2017 [Available from: https://www.northdevonhealth.nhs.uk/have-your-say/current-consultations-and-engagement-opportunities/successregime/acute-services-review/definitions/ accessed 6 March 2019.

6. Department of Health. Older People: National Service Framework for Older People. 2001

7. King's College London, The London School of Economics, Alzheimer's Society. Dementia UK, 2007.

8. Prince MJ, Knapp M, Guerchet M, et al. Dementia UK: Update Second edition. second ed. London, 2014.

9. Featherstone K, Northcott A, Bridges J. Routines of resistance: An ethnography of the care of people living with dementia in acute hospital wards and its consequences. *International Journal of Nursing Studies* 2019 doi: https://doi.org/10.1016/j.ijnurstu.2018.12.009

10. Surr CA, Gates C, Irving D, et al. Effective Dementia Education and Training for the Health and Social Care Workforce: A Systematic Review of the Literature. *Review of educational research* 2017;87(5):966-1002. doi: 10.3102/0034654317723305 [published Online First: 2017/07/31]

11. Scerri A, Innes A, Scerri C. Dementia training programmes for staff working in general hospital settings – a systematic review of the literature. *Aging & Mental Health* 2017;21(8):783-96. doi: 10.1080/13607863.2016.1231170

12. Department of Health. Delivering high quality, effective, compassionate care: Developing the right people with the right skills and the right values. A mandate from the Government to Health Education England: April 2013 to March 2015. London: Crown, 2013.

13. Department of Health. Delivering high quality, effective, compassionate care: Developing the right people with the right skills and the right values. London: Crown, 2014.

For peer review only

For peer review only - http://bmjopen.bmj.com/site/about/guidelines.xhtml

14. CQC. Cracks in the Pathway, 2014-People’s experiences of dementia care as they move between care homes and hospitals. London, 2014.

15. Spector A, Orrell M, Goyder J. A systematic review of staff training interventions to reduce the behavioural and psychological symptoms of dementia. *Ageing Research Reviews* 2013;12(1):354-64. doi: http://dx.doi.org/10.1016/j.arr.2012.06.005

16. Rogers PJ. Using Programme Theory to Evaluate Complicated and Complex Aspects of Interventions. *Evaluation* 2008;14(1):29-48. doi: 10.1177/1356389007084674

17. Woods MD, Kirk MD, Agarwal MS, et al. Vulnerable groups and access to health care: a critical interpretive review. *National Coordinating Centre NHS Service Delivery Organ RD (NCCSDO) Retrieved May* 2005;27:2012.

18. Ahmed F, Morbey H, Harding A, et al. A critical interpretive synthesis (CIS) review of the current evidence base for dementia training in hospitals. *PROSPERO* 2018. http://www.crd.york.ac.uk/PROSPERO/display_record.php?ID=CRD42018088625 (accessed 14 February 2018).

19. CQC. State of health care and social care in England in 2012/13. Technical Annex 3. London: The Stationery Office 2013.

20. Sommerlad A, Perera G, Singh-Manoux A, et al. Accuracy of general hospital dementia diagnoses in England: Sensitivity, specificity, and predictors of diagnostic accuracy 2008-2016. *Alzheimer's & dementia : the journal of the Alzheimer's Association* 2018;14(7):933-43. doi: 10.1016/j.jalz.2018.02.012 [published Online First: 2018/04/29]

21. Carolan CM, Forbat L, Smith A. Developing the DESCARTE Model: The Design of Case Study Research in Health Care. *Qualitative Health Research* 2016;26(5):626-39. doi: 10.1177/1049732315602488

22. Walshe C. The evaluation of complex interventions in palliative care: An exploration of the potential of case study research strategies. *Palliative Med* 2011;25(8):774-81. doi: 10.1177/0269216311419883

23. Morgan SJ, Pullon SR, Macdonald LM, et al. Case Study Observational Research: A Framework for Conducting Case Study Research Where Observation Data Are the Focus. *Qual Health Res* 2016;27(7):1060-68. doi: 10.1177/1049732316649160

24. Pawson R, Tilley N. Realistic Evaluation. . London: Sage 1997.

25. Clarke DJ, Godfrey M, Hawkins R, et al. Implementing a training intervention to support caregivers after stroke: a process evaluation examining the initiation and embedding of programme change. *Implementation Science* 2013;8(1):96. doi: 10.1186/1748-5908-8-96

26. Robson C. Real World Research,. Oxford: Blackwells 1995.

27. Edbrooke-Childs J, Hayes J, Sharples E, et al. Development of the Huddle Observation Tool for structured case management discussions to improve situation awareness on inpatient clinical wards. *BMJ quality & safety* 2018;27(5):365-72. doi: 10.1136/bmjqs-2017-006513 [published Online First: 2017/09/21]

For peer review only - http://bmjopen.bmj.com/site/about/guidelines.xhtml

28. Braun V, Clarke V. Using thematic analysis in psychology. *Qualitative Research in Psychology* 2006;3(2):77-101. doi: 10.1191/1478088706qp063oa

29. Gove D, Diaz-Ponce A, Georges J, et al. Alzheimer Europe's position on involving people with dementia in research through PPI (patient and public involvement). *Aging Ment Health* 2018;22(6):723-29. doi: 10.1080/13607863.2017.1317334 [published Online First: 2017/05/18]

30. Swarbrick CM, Doors O, Educate, et al. Visioning change: Co-producing a model of involvement and engagement in research (Innovative Practice). *Dementia (London, England)* 2016 doi: 10.1177/1471301216674559 [published Online First: 2016/10/19]

31. Morbey H, Harding AJE, Swarbrick C, et al. Involving people living with dementia in research: an accessible modified Delphi survey for core outcome set development. *Trials* 2019;20(1):12. doi: 10.1186/s13063-018-3069-6

Acknowledgements

This work forms part of the Economic and Social Research Council (ESRC) and the National Institute for Health Research (NIHR) Neighbourhoods and Dementia mixed methods study [https://sites.manchester.ac.uk/neighbourhoods-and-dementia/] under grant: ES/L001772/1. This paper is taken from Work Programme 5, Developing the evidence base for evaluating dementia training in NHS hospitals (DEMTRAIN). Our special thanks to Garstang Memory Café, Scottish Dementia Working Group, all of our co-researchers who have helped us, and continue to be an integral part of the DEMTRAIN study. We thank the participants and all who helped us in the study, especially the DEMTRAIN Advisory Group, Dr Carol Opdebeeck, Manchester Metropolitan University, and Marie Crane, Research Co-ordinator.

Authors' contributions:

SR, DR, LD and JK designed the DEMTRAIN study outline submitted to NIHR/ESRC. FA is the lead researcher on the study and led on developing the protocol for this publication. AH and HM are current researchers on the study. SR is the principal investigator for this work programme. JK is the chief investigator for all eight of the Neighbourhoods and Dementia study work programmes. DR, CS, LD, MH, FH, RE, IL, SB, and AB are part of the wider team and provide wider expertise. All authors contributed to the development of the study protocol and this publication.

Funding Statement

This study was funded jointly by the Economic and Social Research Council (ESRC) and the National Institute for Health Research (NIHR) under grant: ES/L001772/1. The ESRC is part of UK Research and Innovation. The views expressed are those of the author(s) and not necessarily those of the ESRC, UKRI, NHS, the NIHR or the Department of Health and Social Care.

For peer review only

Competing interests statement:

None

Data sharing statement

No unpublished data from the phase 2 cohort study are available. For the other phases, some data may be obtained from the corresponding author on reasonable request.

Word count: **4802 or 5046 (including table 1)**

List of figures

Figure 1: DEMTRAIN integration of co-researcher involvement, study phases, data collection and findings

Figure2: Recruitment process for acute NHS hospitals taking part in the DEMTRAIN Hospital Organisational Survey of dementia training and care in England

Figure 3: Proposed data collection structure in each of the six hospital

For peer review only

Figure 1: DEMTRAIN integration of co-researcher involvement, study phases, data collection and findings

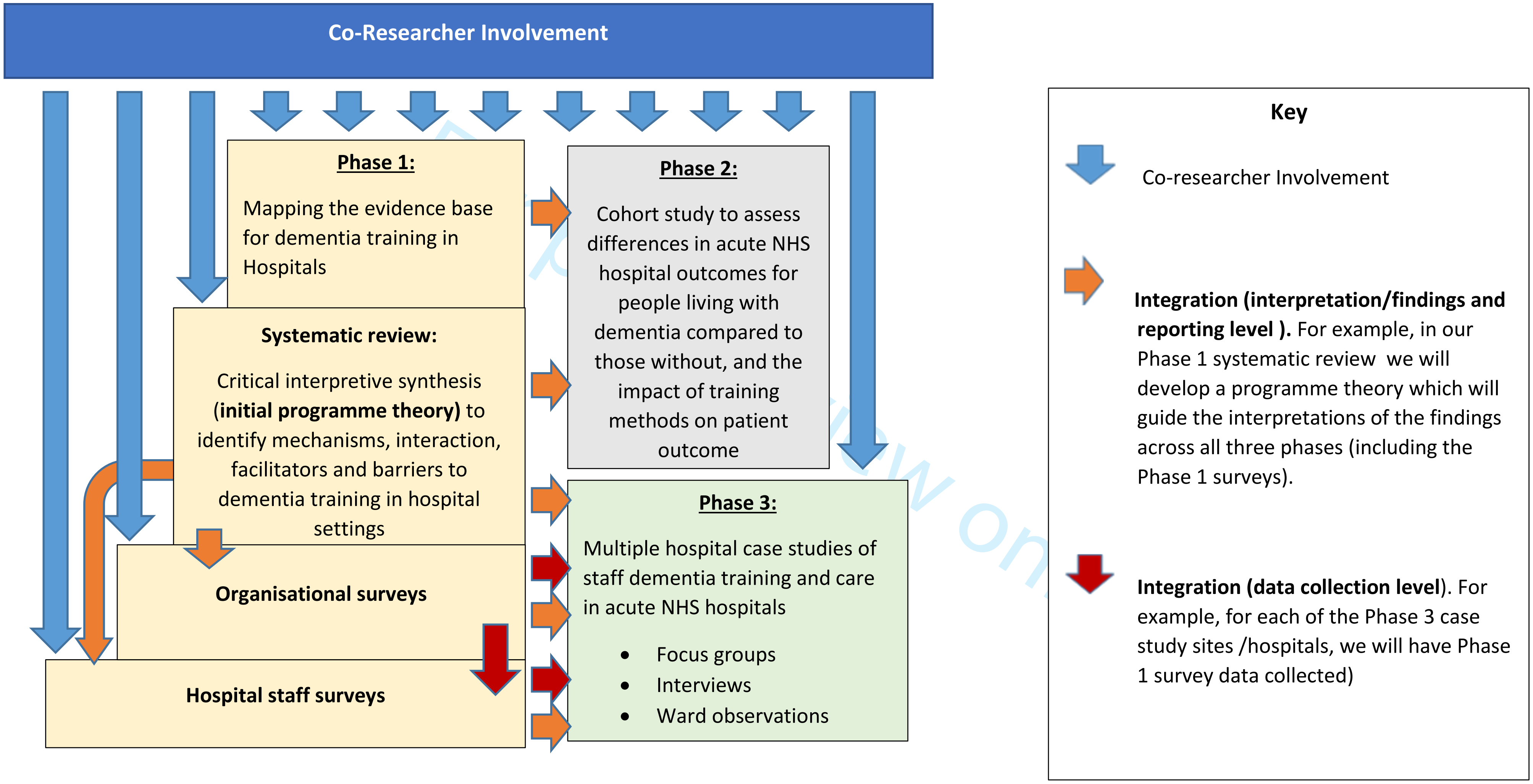

Figure 2: Recruitment process for acute NHS hospitals taking part in the DEMTRAIN Hospital Organisational Survey of dementia training and care in England

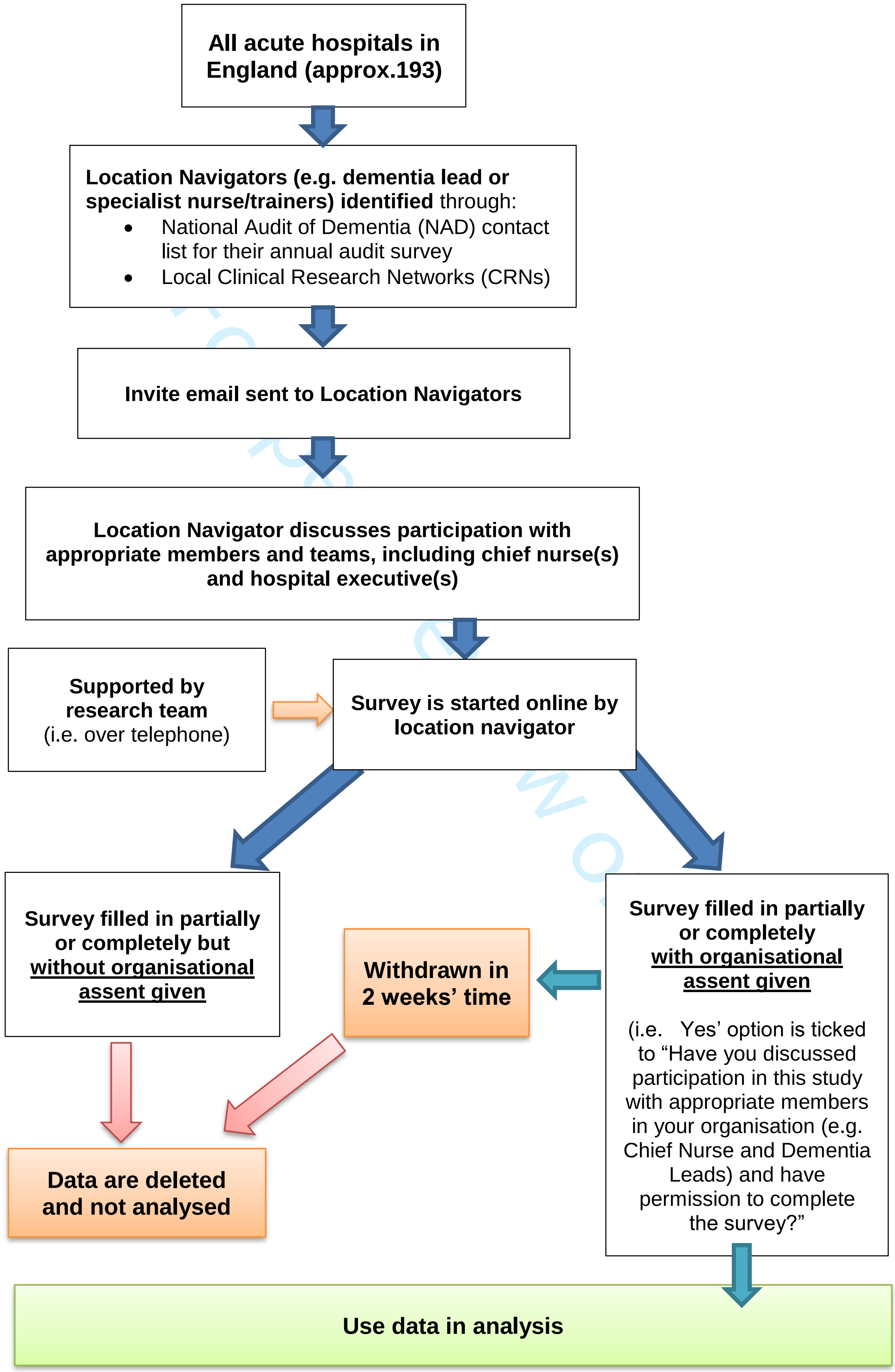

Figure 3: Proposed data collection structure in each of the six hospitals

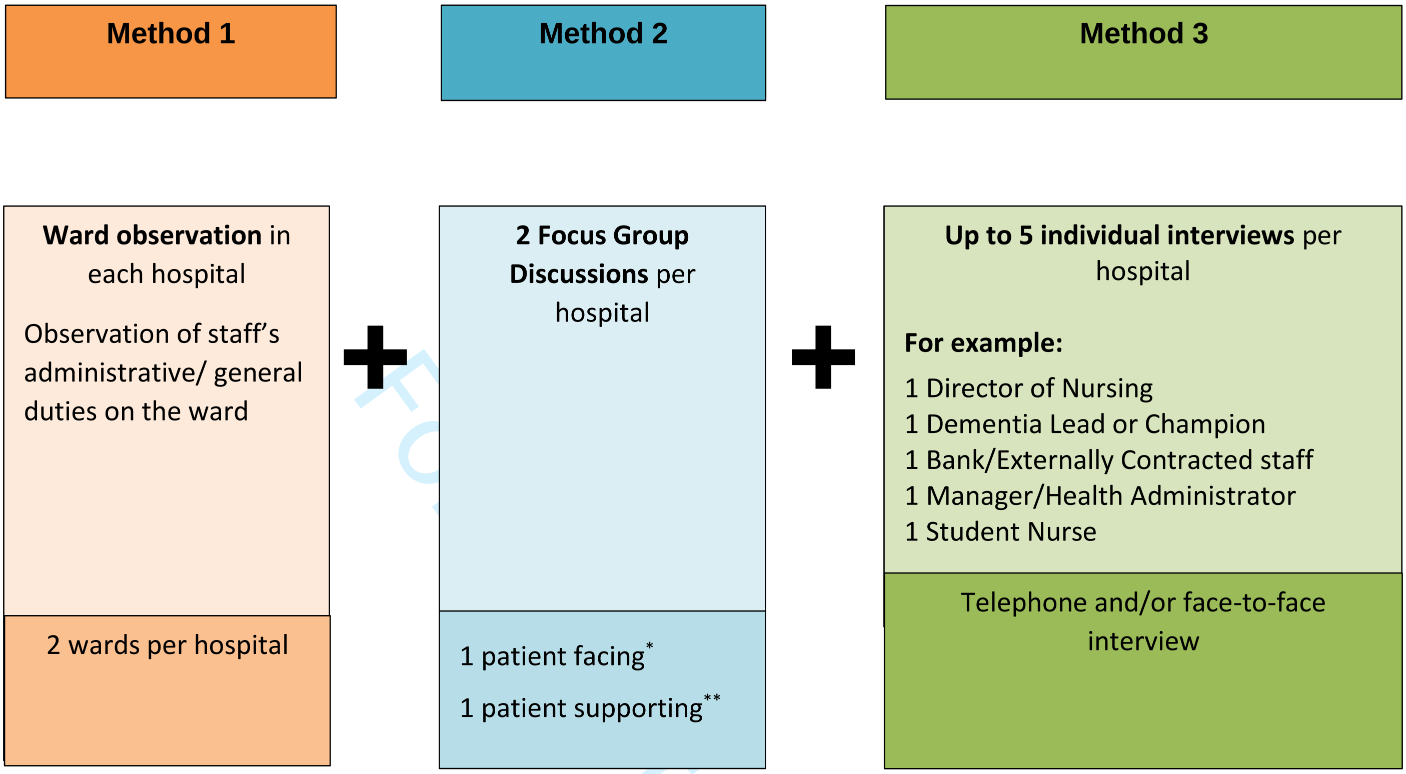

*Patient facing: Doctors, nurses, health care assistants, allied health professionals, including outpatient, A&E, X-rays, dietitians, frailty ward staff, student nurses.

**Patient supporting: Social care, voluntary (community and hospital), discharge staff, patient advice and liaison service staff, car park, & security staff, porters, and other domestic and support staffs

STANDARD PROTOCOL ITEMS: RECOMMENDATIONS FOR INTERVENTIONAL TRIALS

SPIRIT 2013 Checklist: Recommended items to address in a clinical trial protocol and related documents*

Section/item	Item No	Description	Addressed on page number
**Administrative information**			
Title	1	Descriptive title identifying the study design, population, interventions, and, if applicable, trial acronym	4
Trial registration	2a	Trial identifier and registry name. If not yet registered, name of intended registry	NA (page 5 Prospero reg)
	2b	All items from the World Health Organization Trial Registration Data Set	NA
Protocol version	3	Date and version identifier	NA
Funding	4	Sources and types of financial, material, and other support	17
Roles and responsibilities	5a	Names, affiliations, and roles of protocol contributors	17
	5b	Name and contact information for the trial sponsor	17
	5c	Role of study sponsor and funders, if any, in study design; collection, management, analysis, and interpretation of data; writing of the report; and the decision to submit the report for publication, including whether they will have ultimate authority over any of these activities	17
	5d	Composition, roles, and responsibilities of the coordinating centre, steering committee, endpoint adjudication committee, data management team, and other individuals or groups overseeing the trial, if applicable (see Item 21a for data monitoring committee)	17

For peer review only - http://bmjopen.bmj.com/site/about/guidelines.xhtml

Introduction

Background and rationale	6a	Description of research question and justification for undertaking the trial, including summary of relevant studies (published and unpublished) examining benefits and harms for each intervention	4
	6b	Explanation for choice of comparators	NA
Objectives	7	Specific objectives or hypotheses	5, 7 and 8
Trial design	8	Description of trial design including type of trial (eg, parallel group, crossover, factorial, single group), allocation ratio, and framework (eg, superiority, equivalence, noninferiority, exploratory)	5, 7 and 8

Methods: Participants, interventions, and outcomes

Study setting	9	Description of study settings (eg, community clinic, academic hospital) and list of countries where data will be collected. Reference to where list of study sites can be obtained	5-10
Eligibility criteria	10	Inclusion and exclusion criteria for participants. If applicable, eligibility criteria for study centres and individuals who will perform the interventions (eg, surgeons, psychotherapists)	5-10
Interventions	11a	Interventions for each group with sufficient detail to allow replication, including how and when they will be administered	NA
	11b	Criteria for discontinuing or modifying allocated interventions for a given trial participant (eg, drug dose change in response to harms, participant request, or improving/worsening disease)	NA
	11c	Strategies to improve adherence to intervention protocols, and any procedures for monitoring adherence (eg, drug tablet return, laboratory tests)	NA
	11d	Relevant concomitant care and interventions that are permitted or prohibited during the trial	NA
Outcomes	12	Primary, secondary, and other outcomes, including the specific measurement variable (eg, systolic blood pressure), analysis metric (eg, change from baseline, final value, time to event), method of aggregation (eg, median, proportion), and time point for each outcome. Explanation of the clinical relevance of chosen efficacy and harm outcomes is strongly recommended	NA
Participant timeline	13	Time schedule of enrolment, interventions (including any run-ins and washouts), assessments, and visits for participants. A schematic diagram is highly recommended (see Figure)	NA

For peer review only - http://bmjopen.bmj.com/site/about/guidelines.xhtml

Sample size	14	Estimated number of participants needed to achieve study objectives and how it was determined, including clinical and statistical assumptions supporting any sample size calculations	5-10
Recruitment	15	Strategies for achieving adequate participant enrolment to reach target sample size	5-7, and 12-13
**Methods: Assignment of interventions (for controlled trials)**			
Allocation:			
Sequence generation	16a	Method of generating the allocation sequence (eg, computer-generated random numbers), and list of any factors for stratification. To reduce predictability of a random sequence, details of any planned restriction (eg, blocking) should be provided in a separate document that is unavailable to those who enrol participants or assign interventions	NA
Allocation concealment mechanism	16b	Mechanism of implementing the allocation sequence (eg, central telephone; sequentially numbered, opaque, sealed envelopes), describing any steps to conceal the sequence until interventions are assigned	NA
Implementation	16c	Who will generate the allocation sequence, who will enrol participants, and who will assign participants to interventions	5-10
Blinding (masking)	17a	Who will be blinded after assignment to interventions (eg, trial participants, care providers, outcome assessors, data analysts), and how	NA
	17b	If blinded, circumstances under which unblinding is permissible, and procedure for revealing a participant’s allocated intervention during the trial	NA
**Methods: Data collection, management, and analysis**			
Data collection methods	18a	Plans for assessment and collection of outcome, baseline, and other trial data, including any related processes to promote data quality (eg, duplicate measurements, training of assessors) and a description of study instruments (eg, questionnaires, laboratory tests) along with their reliability and validity, if known. Reference to where data collection forms can be found, if not in the protocol	NA
	18b	Plans to promote participant retention and complete follow-up, including list of any outcome data to be collected for participants who discontinue or deviate from intervention protocols	5-7, and 12-13

Data management	19	Plans for data entry, coding, security, and storage, including any related processes to promote data quality (eg, double data entry; range checks for data values). Reference to where details of data management procedures can be found, if not in the protocol	5-6 and 12-13
Statistical methods	20a	Statistical methods for analysing primary and secondary outcomes. Reference to where other details of the statistical analysis plan can be found, if not in the protocol	7-8, and 10-11
	20b	Methods for any additional analyses (eg, subgroup and adjusted analyses)	NA
	20c	Definition of analysis population relating to protocol non-adherence (eg, as randomised analysis), and any statistical methods to handle missing data (eg, multiple imputation)	NA
**Methods: Monitoring**			
Data monitoring	21a	Composition of data monitoring committee (DMC); summary of its role and reporting structure; statement of whether it is independent from the sponsor and competing interests; and reference to where further details about its charter can be found, if not in the protocol. Alternatively, an explanation of why a DMC is not needed	NA
	21b	Description of any interim analyses and stopping guidelines, including who will have access to these interim results and make the final decision to terminate the trial	NA
Harms	22	Plans for collecting, assessing, reporting, and managing solicited and spontaneously reported adverse events and other unintended effects of trial interventions or trial conduct	NA
Auditing	23	Frequency and procedures for auditing trial conduct, if any, and whether the process will be independent from investigators and the sponsor	NA
**Ethics and dissemination**			
Research ethics approval	24	Plans for seeking research ethics committee/institutional review board (REC/IRB) approval	12
Protocol amendments	25	Plans for communicating important protocol modifications (eg, changes to eligibility criteria, outcomes, analyses) to relevant parties (eg, investigators, REC/IRBs, trial participants, trial registries, journals, regulators)	12-13

Consent or assent	26a	Who will obtain informed consent or assent from potential trial participants or authorised surrogates, and how (see Item 32)	5-10
	26b	Additional consent provisions for collection and use of participant data and biological specimens in ancillary studies, if applicable	NA
Confidentiality	27	How personal information about potential and enrolled participants will be collected, shared, and maintained in order to protect confidentiality before, during, and after the trial	12-13
Declaration of interests	28	Financial and other competing interests for principal investigators for the overall trial and each study site	NA
Access to data	29	Statement of who will have access to the final trial dataset, and disclosure of contractual agreements that limit such access for investigators	NA
Ancillary and post-trial care	30	Provisions, if any, for ancillary and post-trial care, and for compensation to those who suffer harm from trial participation	NA
Dissemination policy	31a	Plans for investigators and sponsor to communicate trial results to participants, healthcare professionals, the public, and other relevant groups (eg, via publication, reporting in results databases, or other data sharing arrangements), including any publication restrictions	2, 12, 14
	31b	Authorship eligibility guidelines and any intended use of professional writers	17
	31c	Plans, if any, for granting public access to the full protocol, participant-level dataset, and statistical code	NA
**Appendices**			
Informed consent materials	32	Model consent form and other related documentation given to participants and authorised surrogates	NA
Biological specimens	33	Plans for collection, laboratory evaluation, and storage of biological specimens for genetic or molecular analysis in the current trial and for future use in ancillary studies, if applicable	NA

*It is strongly recommended that this checklist be read in conjunction with the SPIRIT 2013 Explanation & Elaboration for important clarification on the items. Amendments to the protocol should be tracked and dated. The SPIRIT checklist is copyrighted by the SPIRIT Group under the Creative Commons "Attribution-NonCommercial-NoDerivs 3.0 Unported" license.

For peer review only - http://bmjopen.bmj.com/site/about/guidelines.xhtml
